# Perspectives and Challenges of Healthcare Professionals, Patients, and Caregivers Regarding Utilizing Antibiotics and Implementing Antibiotic Stewardship in Healthcare Facilities in Low- and Middle-Income Countries: A Systematic Review of Qualitative Studies

**DOI:** 10.3390/antibiotics15050468

**Published:** 2026-05-05

**Authors:** Bode Ireti Shobayo, Cecilia Stålsby Lundborg, Helena Nordenstedt, Hager Saleh, Tolulope Babawarun, Elizabeth Abisola Oyewole, Mosoka Papa Fallah, Megha Sharma

**Affiliations:** 1 Department of Global Public Health, Karolinska Institutet, 171 76 Stockholm, Sweden; cecilia.stalsby.lundborg@ki.se (C.S.L.); helena.nordenstedt@ki.se (H.N.); hager.saleh@ki.se (H.S.); megha.sharma@ki.se (M.S.); 2 National Public Health Institute of Liberia, Oldest Congo Town, Monrovia P.O. Box 1871, Liberia; 3 Department of Medical Specialties, Danderyd University Hospital, 182 88 Dandery, Sweden; 4 Centre for Population and Reproductive Health, University of Ibadan (CPRH-UI), Ibadan 200285, Nigeria; tolumumuni84@gmail.com; 5 Institute of Child Health, College of Medicine, Faculty of Public Health, University of Ibadan, Ibadan 200285, Nigeria; 6 Africa Centers for Disease Control and Prevention, Addis Ababa P.O. Box 3243, Ethiopia; fallahm@africacdc.org; 7 Department of Pharmacology, Ruxmaniben Deepchand Gardi Medical College, Ujjain 456006, India

**Keywords:** perspectives, challenges, antibiotic resistance, perspectives, challenges, antibiotic stewardship, prescribing and dispensing practices, qualitative evidence synthesis, systematic review, LMICs, healthcare workers, patients, caregivers

## Abstract

**Background:** Antibiotic resistance (ABR) is a critical global health threat, disproportionately affecting low- and middle-income countries (LMICs) where systemic constraints, economic pressures and sociocultural factors drive inappropriate antibiotic use. While quantitative studies describe prevalence patterns, they fail to capture the underlying motivations and contextual barriers influencing prescribing and dispensing behaviors. This systematic review synthesizes qualitative evidence on the perceptions of healthcare professionals, patients, and caregivers regarding antibiotic use and explores the barriers and facilitators for implementing antibiotic stewardship programs in LMIC healthcare settings. **Methods:** We conducted a systematic review following PRISMA 2020 guidelines, based on a registered protocol in PROSPERO ID: CRD42024583749. Searches were performed in Medline, Embase, Cochrane Library, Web of Science, and Google Scholar for English-language studies published between 2014 and 2024. Qualitative and mixed-method studies examining stakeholder perspectives on antibiotic use and antibiotic stewardship implementation in LMICs were included. Studies were excluded if they focused exclusively on pediatric or neonatal populations, veterinary medicine, or quantitative outcomes without qualitative components. The data were analyzed using thematic analysis to identify and categorize codes and identify themes following methodological quality assessment of included studies using the Critical Appraisal Skills Programme Qualitative Studies Checklist by two independent reviewers. **Results:** Out of 2214 studies screened, a total of 119 studies from 33 LMICs were included, encompassing over 4000 participants across hospital, primary care, and community settings. Five interlinked themes emerged: (1) antibiotic use as a pragmatic response to diagnostic uncertainty and resource constraints; (2) financial and commercial drivers shaping prescribing and dispensing practices; (3) the disconnect between knowledge, sociocultural norms, and clinical behavior; (4) multi-level structural and professional barriers to antibiotic stewardship implementation; and (5) the critical role of health system vulnerabilities in perpetuating misuse. **Conclusions:** Inappropriate antibiotic use in LMICs reflects rational adaptations to systemic limitations rather than isolated knowledge gaps. Effective ABS strategies must address structural deficiencies, economic incentives, and sociocultural norms, while integrating context-specific interventions that strengthen health systems and engage all levels of care. The findings should, however, be evaluated in light of the geographic unevenness of the evidence base, the exclusion of non-English and gray literature, and lack of certainty assessments for synthesized themes.

## 1. Introduction

Antimicrobials represent a groundbreaking achievement in medicine, revolutionizing the treatment of infections and consistently preserving millions of lives [1,2]. The emergence of antimicrobial resistance (AMR) has however enabled several human infections to circumvent the efficacy of antimicrobial agents, presenting a substantial risk to public health [3,4]. AMR claimed an estimated 1.3 million lives worldwide in 2019 and by 2050 this number has been predicted to reach 10 million annual deaths [5,6]. It has been demonstrated that antibiotic resistance (ABR) is a key contributor to this growing threat by negatively impacting both clinical and therapeutic outcomes. Repercussions can include treatment failures, the need for new antibiotics, longer hospital stays, higher medical expenses, and increased rates of morbidity and mortality [7,8]. The inappropriate use of antibiotics in human medicine, agriculture, and animal husbandry directly contributes to resistance, exacerbating these consequences [9,10,11]. This dramatic rise in global consumption is predominantly driven by the rapid proliferation, and frequent misuse, of antibiotics within low- and middle-income countries (LMICs) [12]. Healthcare facilities across World Health Organization (WHO) regions in LMICs continue to grapple with inappropriate antibiotic use, highlighting the urgent need for stewardship programs adapted to local contexts [13].

Mitigating AMR necessitates a fundamental transformation in antibiotic utilization, a principle integral to antibiotic stewardship (ABS). ABS denotes organized, systematic measures aimed at fostering the judicious use of antimicrobials to maintain their future efficacy, enhance patient outcomes, and mitigate resistance [14]. Although evidence-based ABS frameworks have been developed and executed in high-income countries, their adaptation to LMIC contexts has been difficult and sometimes unproductive [15]. The factors influencing antibiotic use in these contexts are intricate and context-dependent. Designing successful, practical, and lasting ABS interventions for LMICs necessitates a comprehensive knowledge of local factors from the viewpoints of prescribers, dispensers, patients, and policymakers.

Quantitative studies offer crucial epidemiological data regarding the prevalence and patterns of antibiotic use and resistance in LMICs. However, they are fundamentally constrained in elucidating the intricate, context-dependent motivations, perceptions, and decision-making processes that influence these practices [16]. Quantitative data provide insights into the “what” and “how much,” yet they fall short in elucidating the “why”—the social norms, economic pressures, systemic constraints, and cultural beliefs influencing inappropriate prescribing and dispensing at the point of care [17]. Qualitative research is thus essential for exploring the lived experiences and underlying rationales of healthcare providers, caregivers, patients, and policymakers, elucidating the reasoning behind quantitative data and identifying critical barriers and facilitators to behavior change [18,19].

Despite numerous qualitative studies and region-specific reviews investigating the determinants of antibiotic misuse in LMICs, current synthesis remains fragmented, typically focusing on isolated contexts, stakeholder categories, or narrow knowledge gaps. This geographic and thematic fragmentation produces valuable but localized insights that are difficult to translate into broadly applicable policy guidance [20]. Crucially, existing reviews do not capture the interplay of structural, economic, and sociocultural elements across the continuum of care. This underscores a significant deficiency; the absence of a comprehensive, high-level synthesis that integrates scattered qualitative evidence into a cohesive, actionable approach capable of guiding contextually relevant yet scalable antibiotic stewardship policies and interventions. Beyond consolidating existing qualitative findings, this review makes a conceptual contribution by reframing inappropriate antibiotic use in LMICs as a rational, system-level adaptation to structural vulnerability, rather than a deviation driven primarily by knowledge deficits or individual malpractice. By integrating evidence across multiple stakeholders and care settings, the synthesis elucidates how diagnostic scarcity, market incentives, sociocultural expectations, and governance weakness interact to produce predictable patterns of antibiotic use.

Therefore, this systematic review synthesizes qualitative components of qualitative and mixed-method evidence on antibiotic use and stewardship in LMICs to generate a system-level understanding of how structural constraints, economic incentives, and sociocultural expectations jointly shape prescribing, dispensing, and consumption practices across formal and informal care settings. By consolidating fragmented qualitative evidence across regions and stakeholder groups, this review aims to inform context-sensitive and differentiated antibiotic stewardship policies that are responsive to real-world conditions in LMIC health systems.

## 2. Results

### 2.1. Characteristics of Studies

Evidence synthesized from the 119 studies conducted between 2014 and 2024 in this systematic review was geographically diverse, with the greatest concentration in South Asia and Sub-Saharan Africa. The largest number of studies originated from India (*n* = 13), Pakistan (*n* = 12), and Ethiopia (*n* = 7).

Across the studies, the emphasis was on exploring antimicrobial perceptions, attitudes, practices, and knowledge. Others also examined prescribing behaviors, community perspectives—particularly how community members purchase medicines from pharmacies—and the broader challenges of implementing ABS programs in LMICs. Sample sizes ranged from small groups of 12–84 individuals in country-specific qualitative interviews to over two hundred for multi-country studies. The studies were conducted in diverse healthcare settings, including tertiary and secondary hospitals, community and retail pharmacies, primary healthcare clinics, and various outpatient and informal care contexts.

The included studies employed predominantly qualitative methodologies (*n* = 85), with a significant number of mixed-method studies (*n* = 35) incorporating qualitative components. The total number of participants across all studies exceeded 4000, representing a broad spectrum of stakeholders: healthcare professionals (physicians, pharmacists, nurses, midwives, laboratory staff), drug dispensers, health managers, policymakers, and community members, including patients and caregivers (Table 1).

**Figure 1:**
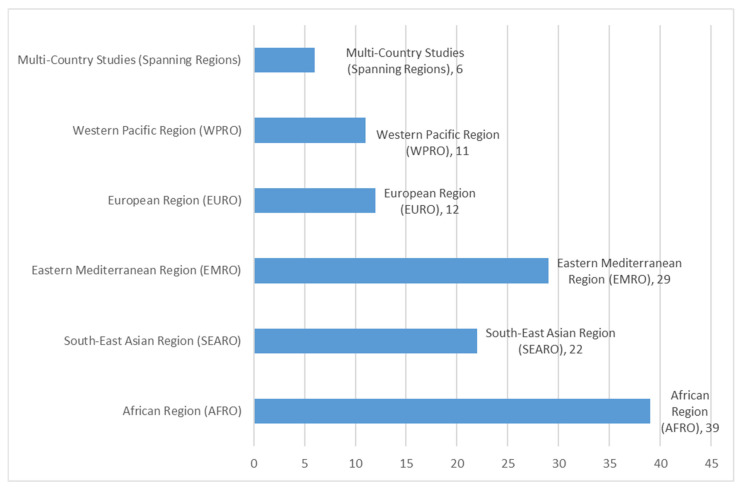
Distribution of studies included by WHO region.

When mapped to WHO regions, most studies were from the African Region (AFR), the Southeast Asia Region (SEAR) and the Eastern Mediterranean Region (EMR) (Figure 1).

### 2.2. Quality Assessment of Included Studies

Based on quality assessment using the CASP tool, the methodological rigor of the included studies was generally high. The mean quality score across the 119 studies was 8.7 out of 10 (SD = 0.97), with scores ranging from 5.5 to 10. The majority of studies (*n =* 95, 80%) scored 8.5 or higher, indicating robust design, appropriate methodology, and clear reporting of findings. Common strengths across high-scoring studies included clear research aims, rigorous data collection, and thorough data analysis. Recurrent limitations among lower-scoring studies (scores < 8) were insufficient description of sampling strategies, lack of reflexivity regarding researcher influence, and inadequate discussion of ethical considerations. No studies were excluded based on quality score alone, as all contributed conceptually relevant data to the thematic synthesis (Table 2).

### 2.3. Thematic Analysis

Through a thematic synthesis of the findings, we identified five overarching, interlinked themes that explain the drivers of antibiotic use and the challenges of implementing ABS in LMICs (Table 3). Taken together, the five themes constitute an integrated explanatory model in which systematic constraints, market forces, sociocultural norms, and governance gaps mutually reinforce one another across formal and informal healthcare settings, shaping antibiotic use practices in predictable ways.

#### 2.3.1. Antibiotic Use as a Pragmatic Response to Systemic and Structural Constraints

The synthesis strongly indicated that inappropriate antibiotic prescribing and dispensing are often not acts of individual negligence but rational, pragmatic responses to profound systemic constraints. The lack of access to reliable and timely diagnostic microbiology was a pervasive barrier cited across hospital and community settings (pharmacies). This diagnostic uncertainty led to empirical prescribing, often with broad-spectrum antibiotics perceived as a safer, more effective general solution [49,91,116]. Physicians in Cambodia explicitly stated a preference for broad-spectrum agents because “it can shoot better” [116]. In parallel, extreme workload, understaffing, and time pressures compelled healthcare providers to use antibiotics as a perceived “quick fix” to manage high patient volumes and clinical uncertainty [54,93,124]. This was compounded by frequent stock-outs of essential antibiotics in public facilities, forcing prescribers to substitute available, often inappropriate agents [62,106].

#### 2.3.2. Financial, Commercial, Socioeconomic, and Cultural Drivers of Misuse

Economic imperatives emerged as a powerful, multi-layered driver of antibiotic misuse. At the provider level, antibiotic sales were directly linked to livelihood and revenue, particularly for informal providers, pharmacy attendants, and private practices. Studies from India, Bangladesh, and Vietnam detailed how the commercial need to sustain a business overrode regulatory and ethical guidelines [61,71,112]. In the private sector, patient retention and satisfaction pressures led to prescribing on demand, with physicians fearing loss of clientele to competitors if they did not comply with patient expectations for an antibiotic [45,54,139]. From the patient perspective, poverty and catastrophic health expenditures drove behaviors such as purchasing partial courses, cheaper subtherapeutic doses, or demanding “strong” injections to maximize perceived value from limited resources [66,82,110].

#### 2.3.3. The Disconnect Between Knowledge, Sociocultural Norms, and Practice

While gaps in technical knowledge regarding guidelines, e.g., for surgical prophylaxis, [82,101,104] and antibiotic resistance were identified [27,140], the findings consistently highlighted that knowledge alone was insufficient to change practice. A critical barrier was the perception of AMR as a distant, abstract threat rather than an immediate clinical concern. Healthcare providers, especially in crisis settings, prioritized treating the patient at hand over the long-term ecological impact of antibiotic use [29,57,94]. Furthermore, deep-seated sociocultural beliefs positioned antibiotics as “strong” and essential medicines for a rapid recovery [47,66,141]. This created immense social pressure on providers to prescribe, framed as a necessity to maintain patient trust and fulfill perceived therapeutic expectations [45,124,139]. This often resulted in a “clinician–client complicity” where both parties colluded in antibiotic use to satisfy these deeply held beliefs [76].

#### 2.3.4. Fragmented Stewardship: Organizational Gaps, Role Ambiguity, and Professional Tensions

Studies focusing on ABS programs revealed a complex array of implementation barriers. Structural and organizational challenges were fundamental, including a lack of dedicated funding, formalized multidisciplinary ABS teams, protected time for ABS activities, and supportive information technology systems [62,87,99]. Electronic prescribing systems, when present, could even introduce new errors [60]. Professional hierarchies and inter-professional tensions also posed significant hurdles. ABS initiatives, often led by pharmacists or microbiologists, were sometimes perceived as challenging physician autonomy and clinical judgment, leading to resistance [30,67,127]. Conversely, the potential of nurses as key stewards was frequently under-realized. While nurses expressed willingness to engage in roles such as prompting antibiotic reviews and patient education, they faced barriers including lack of formal authority, rigid hierarchies, and undefined responsibilities within ABS frameworks [107,137,142]. Finally, weak regulatory enforcement was ubiquitously reported, particularly regarding the non-prescription sale of antibiotics in pharmacies, which was driven by commercial pressure and a lack of effective oversight [31,39,59,128].

#### 2.3.5. Informal and Market-Driven Antibiotic Use Beyond Formal Stewardship Systems

The synthesis underscored that in several countries, the informal and private sectors are the first and often only point of care for a majority of populations in many LMICs, making them critical loci for antibiotic access and misuse. Informal providers, drug shop attendants, and unregulated pharmacies operated with significant autonomy, their practices primarily shaped by customer demand, commercial survival, and frequently, a lack of formal training [42,61,71,84]. These sectors were characterized by a complex supply network that often circumvented formal regulatory channels [112]. The evidence clearly indicates that national ABS policies and regulations focusing solely on the public and formal private sectors are ineffective, as they fail to address the dominant channels of antibiotic provision in many communities [55,82,100].

## 3. Discussion

The principal conceptual contribution of this synthesis is the demonstration that inappropriate antibiotic use in LMICs is best understood as a pragmatic response to systemic vulnerability, rather than as an isolated failure of knowledge, ethics, or compliance. Our synthesis transcends the mere identification of impediments to prescription, instead seeing provider conduct as a reasonable reaction to a milieu of shortage and uncertainty [143,144]. This perspective contrasts with earlier narratives that often implicitly attributed overuse to deficits in knowledge or professional ethics [1,145]. While such deficits exist, our findings resonate with recent health systems research emphasizing the structural vulnerability of both patients and providers in resource-limited settings, where clinical decisions are heavily shaped by external forces beyond individual control. For instance, the preference for broad-spectrum antibiotics as an all-encompassing solution due to diagnostic gaps [146] echoes findings from studies on clinical decision-making under uncertainty in both high- and low-income settings, though the consequences are magnified in LMICs where diagnostic alternatives are virtually nonexistent [147,148]. Likewise, the employment of antibiotics as a time-efficient “quick fix” amidst overwhelming workloads parallels findings on physician burnout and heuristic-based prescribing; however, this issue is exacerbated in low- and middle-income countries (LMICs) by high patient-to-provider ratios that render comprehensive evaluation practically unfeasible [149,150]. The findings suggest that antibiotic stewardship policies in LMICs must emphasize systemic reforms, including enhancing access to point-of-care diagnostics, rectifying medicine supply-chain deficiencies, and addressing financial incentives associated with antibiotic sales, while adapting interventions for various care settings such as hospitals, community pharmacies, and informal providers.

When compared to other systematic reviews of qualitative evidence on antibiotic use, our findings on systemic drivers corroborate key themes. Reviews focusing on specific regions, such as Sub-Saharan Africa or Southeast Asia, have consistently highlighted diagnostic limitations and workload pressures as critical facilitators of inappropriate use [151]. Our global synthesis presents a compelling and universal argument that these issues are not isolated or localized challenges, but rather basic features of under-resourced health systems that consistently influence behavior across continents. Furthermore, our integration of evidence from both formal hospital settings and informal community pharmacies reveals that these constraints operate along a continuum of care. The stock-out of a first-line antibiotic in a public clinic and the subsequent substitution by a provider directly mirrors the community pharmacist’s decision to dispense whatever is on the shelf based on commercial viability rather than guideline appropriateness [152,153]. This highlights that the “pragmatic” logic of availability surpasses professional borders and healthcare sectors, indicating that interventions must address the full therapeutic continuum, from national supply chains to private drug shops, to achieve efficacy [154].

This research identifies financial and commercial imperatives as a primary driver of antibiotic misuse, offering a crucial economic perspective for interpreting prescription and dispensing practices in LMICs. Our findings align closely with political economy assessments of global health, which regard medicine not just as a therapeutic instrument but as a crucial commodity within intricate market systems [155]. The clear connection between antibiotic sales and the financial well-being of providers, especially in informal and private sectors, sharply contrasts with ABS models from high-income nations that presume a distinction between clinical decision-making and direct business incentives [156,157]. This establishes a significant perverse incentive, because the economic rationale of expanding sales and patient churn directly opposes the public health objective of preserving antimicrobials.

This cycle is further intensified by patient-side economics, as poverty drives cost-optimizing behaviors such as acquiring incomplete treatment courses, a rational individual decision that collectively contributes to population-level resistance [158]. Thus, our findings indicates that just clinical or educational treatments are likely to be ineffective unless they address these fundamental market flaws. Effective ABS must incorporate financially astute designs, including the exploration of alternative business models for pharmacies, the separation of provider income from drug sales, and the development of health financing mechanisms that safeguard patients from catastrophic expenses and providers from revenue loss when antibiotics are judiciously withheld [159,160,161].

This synthesis highlights that the disparity between knowledge and practice in the use of antibiotics is not only an informational deficiency but a significant sociocultural gap. Our discovery that AMR is regarded as a somewhat abstract threat, overshadowed by the urgent need to address the presenting patient, corresponds with risk perception theory and significantly contrasts with ABS scenarios in high-income countries, where AMR is frequently characterized as an immediate crisis at both the hospital and patient levels [13,162]. Our review critically illustrates how antibiotics are integrated into a cultural framework of healing, emerging from microbial agents into powerful symbols of strong treatment and physician effectiveness [163]. This cultural capital exerts significant societal pressure on providers, resulting in the clinician and client complicity identified in our findings, a phenomena similarly noted in research concerning injection usage and demands for superfluous procedures [164]. This undermines the fundamental premise of several awareness programs that only presenting the accurate scientific information will change behavior. Interventions must transcend mere knowledge transmission to actively reformulate narratives and social norms. This necessitates community-focused communication that redefines antibiotics as a limited resource rather than a symbol of strength, while equipping healthcare providers with dialog frameworks and social strategies to advocate for non-prescription, therefore maintaining the therapeutic relationship and patient trust.

The implementation challenges faced by ABS programs in LMICs, as summarized in this review, expose a complex, multi-tiered implementation cliff that goes well beyond merely lacking technical guidelines. The structural deficiencies including lack of funding, inadequate staffing, and limited time reflect well-documented obstacles to quality development initiatives within fragile health systems [143,165]. These challenges underscore that ABS cannot function as a standalone program but must be incorporated into comprehensive health system strengthening strategies. Furthermore, socio-professional barriers, including the threat that ABS poses to physician autonomy and the systematic underutilization of nurses, exemplify profoundly rooted power structures within healthcare hierarchies [166]. This finding contrasts with the successful ABS models implemented in high-resource environments, which frequently depend on a culture of interdisciplinary collaboration and the formalization of nurse stewardship roles [167,168]. The concurrent shortcomings in regulatory enforcement, particularly regarding pharmacy sales, underscore a significant governance deficiency wherein policies are documented but not effectively implemented, a prevalent issue observed in studies of law enforcement within LMICs [169,170]. The primary implication is that implementing high-income country ABS blueprints is likely not to prove successful. Instead, context-specific models should be developed that pragmatically address existing hierarchies, such as engaging senior physicians as champions, formally incorporating and empowering nurses and pharmacists within their designated scopes of practice, and combining regulatory strategies with supportive, enabling interventions for pharmacies to mitigate the commercial incentives behind non-prescription sales.

The identification within this review of the informal and private sectors as the primary, yet frequently disregarded, ecosystem for antibiotic distribution constitutes a crucial reconsideration of the AMR challenge in numerous LMICs. The results of this study indicate that the standard model of a formal, regulated healthcare system often represents a minority pathway for treatment. This observation is supported by health utilization research conducted throughout Africa and Asia, which reveals a significant dependence on informal and retail drug outlets for initial healthcare access [171,172]. The practices within this ecosystem, propelled by commercial interests and consumer preferences rather than clinical guidelines, adhere to a profoundly distinct rationale compared to that presupposed by most national ABS policies [173]. This situation results in a significant disconnect between policy and practice, wherein regulatory measures aimed at licensed hospitals and pharmacies are undermined by a parallel, adaptable, and economically driven market operating beyond established governance frameworks [174]. The clear implication is that any ABS strategy which does not actively and innovatively involve the informal and private sectors is inherently deficient. Future interventions should transcend regulatory approaches that rely solely on prohibition; as such strategies frequently encourage clandestine practices, and should, instead, investigate governance models that are inclusive in nature. These potential interventions encompass training and accreditation for informal healthcare providers, establishing economic incentives to encourage drug stores to promote judicious dispensing practices, and developing streamlined stewardship tools that are practical within the high-volume, low-profit environment characteristic of community retail pharmacies.

## 4. Materials and Methods

### 4.1. Protocol Registration

We developed the methods for this study based on the Preferred Reporting Items for Systematic Reviews and Meta-Analyses (PRISMA 2020) guidelines [175] and submitted the protocol to the International Prospective Register of Systematic Reviews (PROSPERO) on 27 August 2024 (review ID: CRD42024583749) [176].

### 4.2. Eligibility Criteria

All articles identified through the search were screened against the following inclusion criteria: (a) reported primary data; (b) presented findings from qualitative research methods (e.g., interviews, focus groups); (c) explored the perspectives of healthcare professionals, patients, and caregivers on antibiotic prescription and dispensing in low- and middle-income countries (LMICs); (d) identified challenges and/or enablers in implementing ABS programs within healthcare facilities in LMICs; and (e) reported data collected across all levels of healthcare facilities.

Due to differences in antibiotic resistance patterns and profiles between children and adults and in prescription standards (e.g., regarding antibiotic duration), the review did not include prescriptions for neonates or children. Papers on the use of antibiotics in veterinary medicine, although a significant contributor to the development of AMR, were also excluded. No studies were excluded based on quality to ensure inclusion of all potentially valuable insights, though quality assessments were used to contextualize the strength and credibility of the findings [177].

### 4.3. Search Strategy and Data Source/Collection

We performed a search of the literature in the following databases: Medline (Ovid), Embase (embase.com), Cochrane Library (Wiley) and Web of Science (Clarivate Analytics). A complementary search was performed in Google Scholar and the first 200 hits were reviewed. The last search was conducted on 15 October 2024. The search strategy was developed in Medline (Ovid) in collaboration with librarians at the Karolinska Institutet University Library. For each search concept, Medical Subject Headings (MeSH-terms) and free-text terms were identified. The search was then translated, in part using Polyglot Search Translator [178], into the other databases. No language restriction was applied, and databases were searched from inception. The strategies were proof-read by another librarian prior to execution (Supplementary Materials).

### 4.4. Study Selection and Screening Process

All the articles identified from the electronic databases were transferred to Covidence (Veritas Health Innovation, Melbourne, Australia) for screening and data management purposes [179]. Duplicates were removed automatically and subsequently confirmed through manual verification. Two independent reviewers (BIS and EAO) screened all titles and abstracts against the predefined eligibility criteria. Each reviewer was blinded to the other’s decisions. Disagreements were resolved through discussions, and if a tiebreaker was needed, a decision was made by consensus. This process was repeated for full-text screening.

**Figure 2:**
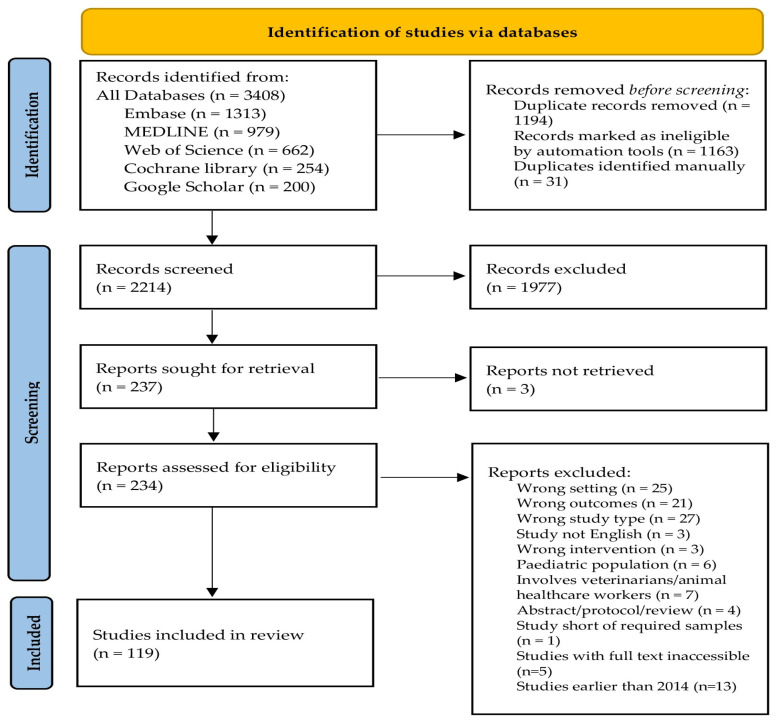
Flowchart of the search strategy following the PRISMA 2020 guidelines for systematic reviews.

The initial search yielded 3408 records. After removing duplicates (*n* = 1194), abstract screening excluded 1977 irrelevant studies. Full-text review of 234 studies resulted in 119 eligible studies. The reasons for exclusion at this stage were documented in Covidence and categorized according to the exclusion criteria: wrong setting (*n* = 25), wrong outcomes (*n* = 21), wrong study type (*n* = 27), non-English-language (*n* = 3), wrong intervention (*n* = 3), pediatric population (*n* = 6), involves veterinarians/animal healthcare workers (*n* = 6), abstract-/protocol-only or review article (*n* = 4), study short of required samples (*n* = 1), studies with full text inaccessible (*n* = 5), and studies earlier than 2014 (*n* = 13). Out of the 119 studies, 89 used qualitative methods and 30 mixed methods. Studies using mixed approaches were allowed to proceed to full-text extraction as long as the qualitative component fit the inclusion criteria and was relevant to the study objectives. Any disagreements during full-text screening were resolved through discussion and consensus. The PRISMA flow diagram below details the selection process (Figure 2).

### 4.5. Outcomes and Data Synthesis

This review sought qualitative findings across two primary outcome domains: (1) stakeholder perspectives on antibiotic use, including attitudes, beliefs, perceptions and experiences of healthcare professionals, patients, and caregivers regarding prescribing, dispensing and consumption practices; and (2) barriers and facilitators to antibiotic stewardship implementation in LMIC healthcare settings. All qualitative findings compatible with these domains were sought from each included study. For mixed-method studies, only qualitative components were extracted. Findings were excluded if they focused exclusively on pediatric populations, veterinary medicine, quantitative prescribing patterns, or stakeholder groups outside the defined scope. The extracted data were subsequently coded and synthesized thematically as described below.

### 4.6. Data Extraction Process

Data were extracted from all included studies to capture key characteristics relevant to the review objectives, including bibliographic information, study setting, study design, data collection methods, participant characteristics (type and number), key findings related to antibiotic use and stewardship, stakeholder perspectives, and reported barriers or facilitators. Two independent reviewers (BIS and EAO) were responsible for data extraction and the data extracted were compared to identify any discrepancies and to ensure the absence of typo errors. Disagreements were resolved through discussion to ensure consistency and accuracy. No automation tools were used for data extraction.

### 4.7. Quality Assessment

The Critical Appraisal Skills Program (CASP) Checklist [180] was used to evaluate the qualitative components of both qualitative and mixed-method studies based on a numerical rating system [180,181]. Two independent reviewers (BIS and EAO) assessed each study working independently. Differences in quality appraisal scores were discussed between reviewers and resolved by consensus before final scores were assigned. The CASP tool utilized contained ten components, including aim clarity, method suitability, design, sampling strategy, data collection, reflexivity, ethical concerns, data analysis, findings, and research value. Each section was assigned one point if it was fully explored in the paper, half a point if it was partially addressed, and zero if it was not addressed. Based on this ranking, the maximum attainable score was 10. There were no studies excluded due to their quality but assessments were used to contextualize the strength and credibility of findings in the synthesis (Table 2). A formal assessment of certainty in the synthesized findings was not performed as this study was designed as an exploratory qualitative evidence synthesis to identify and interpret key themes.

### 4.8. Thematic Analysis and Synthesis

All 119 studies meeting the inclusion criteria were eligible for synthesis; no further restrictions were applied at the synthesis stage. The study characteristics were tabulated to summarize the key features, the methodological quality assessments were tabulated to reflect rigor, and final themes with contributing studies were presented to illustrate analytical findings. Geographic distribution was visualized using a map of WHO regions, and the study selection process was illustrated using a PRISMA flow diagram. Data synthesis was performed using thematic analysis in accordance with a six-phase framework [182]. Given the substantial heterogenicity in study settings, populations, and qualitative methodologies, thematic synthesis focused on identifying conceptual patterns and explanatory mechanisms rather than aggregating context-specific frequencies. The qualitative data extracted from the included studies were reviewed repeatedly to develop familiarity with the data. Initial coding and theme development were led by the first author, drawing inductively from extracted qualitative data. Emerging themes were iteratively refined through comparison with the original study findings and guided by the review objectives. Co-authors contributed to the interpretation and refinement of final themes through critical discussion of thematic structure and coherence. Subsequent codes were constructed inductively to encapsulate key concepts pertinent to the review objectives. Related codes were consolidated to identify potential themes, which were then reviewed and refined to guarantee consistency and relevance across the included studies.

Initial codes were consolidated into broad groups to discern recurring patterns. Patient demand, financial incentives, and poverty were classified as Drivers of Inappropriate Use, whereas knowledge gaps, guideline non-adherence, and misconceptions comprised Knowledge and Awareness Gaps. Structural challenges, such as inadequate regulation, insufficient diagnostics, and resource limitations, were grouped into Systematic and Structural barriers, whereas codes pertaining to professional practice (e.g., improper prescribing, role ambiguity, underutilization of nurses) were categorized as Healthcare Professional Roles and Practices. Informal provider practices and over-the-counter sales were merged into Informal Use Outside Stewardship Systems. The categories were refined into five primary themes that clarify the intricate aspects contributing to antibiotic misuse.

While study selection, data extraction, and quality appraisal were conducted independently by two reviewers with discrepancies resolved by consensus, the thematic synthesis itself was led by a single reviewer, which reflects standard practice in exploratory qualitative syntheses but may introduce interpretive subjectivity.

### 4.9. Data Tabulation and Visualization

The study characteristics were summarized to highlight key characteristics across all included studies (Table 1). Individual study scores were presented using tabulated methodological quality assessments (Table 2). The final themes were included alongside sub-themes, supporting codes, and contributing studies (Table 3). A map was used to display geographic distribution (Figure 1) based on WHO region, and a PRISMA flow diagram was used to depict the study selection procedure (Figure 2). Tables and figures were created using Microsoft Excel and Covidence.

## 5. Conclusions

This review demonstrates that antibiotic misuse and stewardship failures in LMICs are not simply the result of individual knowledge gaps or professional negligence, but rather are deeply embedded within the structural, economic, and sociocultural fabric of under-resourced health systems. Inappropriate use emerges as a pragmatic response to pervasive systemic constraints, including diagnostic uncertainty, workforce shortages, and medication stock-outs, while powerful commercial imperatives and cultural beliefs position antibiotics as both an economic commodity and a symbol of effective care. Furthermore, fragmented health systems, professional hierarchies, weak regulatory enforcement, and a dominant informal sector that operates largely outside formal governance hinder ABS initiatives. These interconnected drivers operate across the continuum of care, from tertiary hospitals to community drug shops, indicating that isolated clinical or educational interventions are insufficient to change behavior at scale.

By conceptualizing antibiotic misuse as a symptom of systemic vulnerability rather than individual behavior, this review underscores the need for stewardship policies that prioritize structural reform, financial realignment and context-specific governance. To effectively address ABR in LMICs, these findings indicate that stewardship efforts must move beyond technical guidelines toward explicitly differentiated, system-level policy responses that target the structural drivers of misuse. In practice, this means prioritizing investment in affordable point-of-care diagnostics to reduce empirical prescribing, implementing financing and regulatory reforms that decouple provider income from antibiotic sales, and designing context-specific communication strategies that reshape public and professional expectations around antibiotics. Importantly, stewardship policies should be tailored to distinct care settings, with hospital-based interventions complemented by inclusive governance, training and incentive structures that meaningfully engage private pharmacies and informal providers. By treating antibiotic misuse as a symptom of broader health system vulnerability rather than isolated behavior, policymakers and practitioners can develop more equitable, sustainable, and effective interventions that protect both individual health and long-term antibiotic effectiveness. Future efforts must adopt an integrated, system-strengthening approach that moves beyond technical guidelines to tackle the root causes of misuse. This requires designing context-sensitive ABS models that align with local realities, such as by developing affordable point-of-care diagnostics, creating alternative financing mechanisms to decouple provider income from antibiotic sales, and reframing public and professional narratives around antibiotics. Critically, stewardship strategies must extend beyond the formal health sector to meaningfully engage informal providers and private pharmacies through inclusive governance, training, and incentive structures. By recognizing antibiotic use as a symptom of broader systemic vulnerabilities, policymakers and practitioners can develop more equitable, sustainable, and effective interventions that protect both individual health and global antibiotic efficacy.

## 6. Strengths and Limitations

This review possesses several notable strengths. It incorporates evidence from 119 qualitative and mixed-method studies undertaken in LMIC contexts, offering a comprehensive overview of the systemic, cultural, and economic factors influencing antibiotic use and barriers to implementing ABS. The review includes insights from healthcare professionals, patients, and caregivers, elucidating the intricacies of decision-making processes in formal health systems and highlighting how contextual factors influence prescription and consumption patterns. Methodological rigor is maintained by following PRISMA guidelines and registering prospectively in PROSPERO, thereby ensuring transparency and reproducibility. Moreover, the use of thematic synthesis supports the cohesive integration of diverse research findings, producing actionable insights and useful themes. Collectively, these attributes make the review essential for guiding context-specific ABS interventions that target underlying structural factors instead of merely isolated behavior patterns.

Nonetheless, some limitations should be acknowledged. Firstly, the evidence base was geographically uneven, with limited representation from Latin America, and only English-language studies included. This was addressed in this synthesis by drawing on 119 studies across 33 LMICs and the consistency of themes across diverse contexts support transferability. Nonetheless, language and publication bias remain a concern. Although only three studies were excluded for being non-English, this restriction risks under-representing qualitative evidence from settings where research is published locally. These barriers reflect broader inequities in global health, where linguistic and publishing constraints shape which perspectives enter the international literature. Future synthesis should therefore adopt deliberate strategies—such as multilingual reviews, collaboration with regional researchers, and inclusion of locally published studies—to ensure more equitable representation of antibiotic use and stewardship experiences worldwide.

Secondly, no sensitivity analysis was conducted to assess robustness. However, all studies were retained for comprehensiveness, and quality was systematically assessed using the CASP tool. This study also did not employ a formal certainty assessment, which may limit theme confidence evaluations. Future reviews with decision-making aims may benefit from the integration of a formal certainty assessment. A single reviewer performed the theme synthesis, which may increase the risk of interpretative bias due to the absence of independent coding or verification. While a systematic and transparent analytic approach was used, future syntheses might benefit from double-coding or reviewer triangulation to enhance credibility. This review also excluded pediatric, neonatal, and veterinary populations, potentially constraining the generalizability of the findings, especially in LMICs where pediatric antibiotic usage and human–animal interactions are substantial contributors to antimicrobial resistance. The findings are mostly relevant to adult human healthcare settings. Future reviews that adopt a One Health or life-course approach to include pediatric and veterinary qualitative evidence could offer a more thorough comprehension of AMR determinants. Lastly, the search was restricted to research published from 2014 to 2024, and gray literature was not included. To minimize bias, a comprehensive search across five databases was conducted, capturing a decade of contemporary evidence.

## Figures and Tables

**Table 1:** Characteristics of the studies included in this review.

No.	Author and Year	Study Setting	Study Title	Number of Participants	Category of Participants	Study Design
1	Abdelaziz et al., 2019 [21]	Egypt (community pharmacies)	“Quality of Community Pharmacy Practice in Antibiotic Self-Medication Encounters: A Simulated Patient Study in Upper Egypt”	150	Community pharmacists	Qualitative
2	Abdel Jalil et al., 2023 [22]	Jordan (teaching hospitals)	“Vancomycin prescribing and therapeutic drug monitoring: Challenges of real clinical practice”	34	Physicians, pharmacists, and nurses	Qualitative
3	Abu Mhadi et al., 2023 [23]	Palestine (primary healthcare clinic)	“Exploring medicines use patterns and practices among the public in the Gaza strip, Palestine: A qualitative study”	26	Patients and relatives	Qualitative
4	Ackers et al., 2020 [24]	Uganda (referral hospital)	“Opportunities and Challenges for Improving Anti-Microbial Stewardship in Low- and Middle-Income Countries; Lessons Learnt from the Maternal Sepsis Intervention in Western Uganda”	25	Nurses, midwives, intern doctors, laboratory technicians and pharmacists	Qualitative
5	Afari-Asiedu et al., 2021 [25]	Ghana	“Stakeholders’ perspectives on training over the counter medicine sellers and Community-based”	10	Health services personnel, including the municipal director of health services, disease control officer, and public health nurses	Qualitative
6	Aika and Enato, 2022 [26]	Nigeria (primary and public healthcare facilities)	“Health care systems administrators perspectives on antimicrobial stewardship and infection prevention and control programs across three healthcare levels: a qualitative study”	14	Hospital managers	Qualitative
7	Akhtar et al., 2020 [27]	Malaysia (tertiary care public hospital)	“Physicians’ Perspective on Prescribing Patterns and Knowledge on Antimicrobial Use and Resistance in Penang, Malaysia: A Qualitative Study”	12	Physicians	Qualitative
8	Alemkere et al., 2023 [28]	Ethiopia (hospitals)	“Optimizing prophylactic antibiotic use among surgery patients in Ethiopian hospitals”	48	Hospital staff	Mixed methods
9	Älgå et al., 2018 [29]	Jordan (hospital)	““Reality rarely looks like the guidelines”: a qualitative study of the challenges hospital-based physicians encounter in war wound management”	11	Physicians	Qualitative
10	Älgå et al., 2018 [29]	Jordan (hospital)	“Perceptions of Healthcare-Associated Infection and Antibiotic Resistance among Physicians Treating Syrian Patients with War-Related Injuries”	10	Physicians	Qualitative
11	Alghamdi et al., 2021 [30]	Saudi Arabia (hospital)	“Antimicrobial Stewardship Program Implementation in a Saudi Medical City: An Exploratory Case Study”	5	Core members of the ASP team (infectious disease consultant, antimicrobial lead pharmacist, clinical pharmacists, consultant clinical microbiologist and infection control consultant)	Mixed methods
12	Alkadhimi et al., 2021 [31]	Iraq (community pharmacies)	“Dispensing of antibiotics in community pharmacy in Iraq: a qualitative study.”	20	Community pharmacists	Qualitative
13	Al Meslamani et al., 2022 [32]	Jordan (hospital)	“Antibiotic prescribing errors generated by the use of an electronic prescribing system in the emergency department: A mixed-method study”	15	Emergency department physicians	Mixed methods
14	Amin et al., 2017 [33]	Egypt (community pharmacies)	“Perspectives of pharmacy staff on dispensing subtherapeutic doses of antibiotics: a theory informed qualitative study”	9	Pharmacists and pharmacy assistants	Qualitative
15	Anwar et al., 2021 [34]	Pakistan (provincial hospital)	“Exploring Nurses’ Perception of Antibiotic Use and Resistance: A Qualitative Inquiry”	15	Nurses	Qualitative
16	Asante et al., 2017 [35]	Ghana (public and private health facilities)	“Knowledge of antibiotic resistance and antibiotic prescription practices among prescribers in the Brong Ahafo Region of Ghana; a cross-sectional study”	33	Prescribers	Mixed methods
17	Atif et al., 2020 [36]	Pakistan (community pharmacies)	“Community pharmacists as antibiotic stewards: A qualitative study exploring the current status of Antibiotic Stewardship Program in Bahawalpur, Pakistan”	15	Community pharmacists	Qualitative
18	Atif et al., 2019 [37]	Pakistan (community pharmacies)	“What drives inappropriate use of antibiotics? A mixed methods study from Bahawalpur, Pakistan”	16	Clients of pharmacies	Mixed methods
19	Atif et al., 2021 [38]	Pakistan (tertiary care public hospitals)	“Antibiotic stewardship program in Pakistan: a multicenter qualitative study exploring medical doctors’ knowledge, perception and practices”	17	Medical doctors	Qualitative
20	Bahnassi [39]	Syria (community pharmacies)	“A qualitative analysis of pharmacists’ attitudes and practices regarding the sale of antibiotics without prescription in Syria”	147	Pharmacists	Qualitative
21	Bahta et al., 2021 [40]	Eritrea (drug retail outlets)	“Determinants of dispensing antibiotics without prescription in Eritrea: a mixed-method qualitative study on pharmacy professionals’ perspective”	30	Pharmacy professionals	Mixed methods
22	Bajwa et al., 2024 [41]	Pakistan (drug inspectors offices)	“Drug Inspector as an antibiotic steward: challenges and recommendations”	17	Drug inspectors	Qualitative
23	Barker et al., 2017 [42]	India (village pharmacies)	“What drives inappropriate antibiotic dispensing? A mixed-methods study of pharmacy employee perspectives in Haryana, India”	24	Village pharmacy staff	Mixed methods
24	Baubie et al., 2019 [43]	India (hospital)	“Evaluating antibiotic stewardship in a tertiary care hospital in Kerala, India: a qualitative interview study”	45	Hospital faculty and staff	Qualitative
25	Belachew et al., 2023 [44]	Ethiopia (community drug retail outlets)	““Handing out non-prescribed antibiotics is storing up trouble for the next generation!” Unpacking multi-stakeholder views of drivers and potential solutions in Ethiopia”	23	Pharmacy professionals	Qualitative
26	Broom and Doron, 2020 [45]	India (public and private hospitals)	“Antimicrobial Resistance, Politics, and Practice in India”	24	Doctors	Qualitative
27	Broom et al., 2021 [46]	India (hospitals and pharmacies)	“Antimicrobial overuse in India: A symptom of broader societal issues including resource limitations and financial pressures”	30	Doctors and community pharmacists	Qualitative
28	Cantarero-Arevalo et al., 2022 [47]	Russia (hospitals)	“A Qualitative Analysis of the Culture of Antibiotic Use for Upper Respiratory Tract Infections Among Patients in Northwest Russia”	55	Patients	Qualitative
29	Chansamouth et al., 2024 [48]	Laos (hospital)	“Understanding hospital antimicrobial prescribing decisions and determinants of uptake of new local antimicrobial prescribing guidelines in Laos”	16	Antibiotic prescribers	Qualitative
30	Chukwu et al., 2024 [49]	Nigeria (healthcare facilities)	“Implementation of antimicrobial stewardship programs: A study of prescribers’ perspective of facilitators and barriers”	25	Antibiotic prescribers	Mixed methods
31	Darj et al., 2020 [50]	Bangladesh (pharmacies)	“Pharmacists’ perception of their challenges at work, focusing on antimicrobial resistance: a qualitative study from Bangladesh”	24	Retail pharmacists	Qualitative
32	Di et al., 2022 [51]	Vietnam (hospitals)	“Physician’s Perspectives on Factors Influencing Antibiotic Resistance: A Qualitative Study in Vietnam”	34	Physicians	Qualitative
33	Dillip et al., 2015 [52]	Tanzania (drug-dispensing outlets)	“What motivates antibiotic dispensing in accredited drug dispensing outlets in Tanzania? A qualitative study”	84	Drug-dispensing outlet owners and dispensers	Qualitative
34	Dohou et al., 2022 [53]	Benin (hospitals)	“Healthcare Professionals’ Knowledge and Beliefs on Antibiotic Prophylaxis in Cesarean Section: A Mixed-Methods Study in Benin”	19	Doctors and nurses	Qualitative
35	Dooling et al., 2014 [54]	Egypt (hospitals and pharmacies)	“Understanding Antibiotic Use in Minya District, Egypt: Physician and Pharmacist Prescribing and the Factors Influencing Their Practices”	40	Physicians and pharmacists	Mixed methods
36	Do et al., 2021 [55]	Mozambique, Ghana, South Africa, Bangladesh, Vietnam, and Thailand	“Community-based antibiotic access and use in six low-income and middle-income countries: a mixed-method approach”	259	Antibiotic suppliers and community members	Mixed methods
37	Edessa et al., 2024 [56]	Ethiopia (pharmacies)	“Drug providers’ perspectives on antibiotic misuse practices in eastern Ethiopia”	15	Drug providers	Qualitative
38	Eibs et al., 2020 [57]	Guinea-Bissau, Central African Republic (CAR), Democratic Republic of Congo (DRC) and Sudan (hospitals)	“Qualitative study of antibiotic prescription patterns and associated drivers in Sudan, Guinea-Bissau, Central African Republic and Democratic Republic of Congo”	384	Prescribers and community members	Qualitative
39	Esfandiari et al., 2018 [58]	Iran (ministry of health offices, medical universities and hospitals)	“Eliminating Healthcare-Associated Infections in Iran: A Qualitative Study to Explore Stakeholders’ Views”	24	Healthcare workers, technical officers and policymakers	Qualitative
40	Farooqui et al., 2023 [59]	Pakistan (tertiary care hospital)	“Hospital Pharmacists’ Viewpoint on Quality Use of Antibiotics and Resistance: A Qualitative Exploration from a Tertiary Care Hospital of Quetta City, Pakistan”	12	Pharmacists	Qualitative
41	Foster and Bandawe, 2014 [60]	Malawi (pharmacies)	“How much do patients in Blantyre, Malawi know about antibiotics and other prescription only medicines?”	54	Patients	Mixed methods
42	Gautham et al., 2021 [61]	India (various settings including pharmacies)	“What are the challenges for antibiotic stewardship at the community level? An analysis of the drivers of antibiotic provision by informal healthcare providers in rural India”	47	Pharmaceutical sales representatives, managers and wholesalers/retailers; medically qualified private and public doctors, and health and regulatory officials and community members	Mixed methods
43	Gebretekle et al., 2018 [62]	Ethiopia (specialized hospitals)	“Opportunities and barriers to implementing antibiotic stewardship in low and middle-income countries: Lessons from a mixed-methods study in a tertiary care hospital in Ethiopia”	35	Physicians and pharmacists	Mixed methods
44	Gebretekle and Serbessa, 2016 [63]	Ethiopia (community pharmacies)	“Exploration of over the counter sales of antibiotics in community pharmacies of Addis Ababa, Ethiopia: pharmacy professionals’ perspective”	5	Pharmacy professionals	Qualitative
45	Ghiga et al., 2023 [64]	Romania (online)	“Family doctors’ roles and perceptions on antibiotic consumption and antibiotic resistance in Romania: a qualitative study”	12	Doctors	Qualitative
46	Ghiga and Lundborg, 2016 [65]	Romania (pharmacies)	“‘Struggling to be a defender of health’—a qualitative study on the pharmacists’ perceptions of their role in antibiotic consumption and antibiotic resistance in Romania”	18	Pharmacists	Qualitative
47	Green et al., 2023 [66]	Kenya, Tanzania, Uganda (healthcare facilities)	“The role of multidimensional poverty in antibiotic misuse: a mixed-methods study of self-medication and non-adherence in Kenya, Tanzania, and Uganda”	126	Patients and Community members	Mixed methods
48	Hayat et al., 2019 [67]	Pakistan (tertiary care public hospitals)	“Perspective of Pakistani Physicians towards Hospital Antimicrobial Stewardship Programs: A Multisite Exploratory Qualitative Study”	22	Physicians	Qualitative
49	Horter et al., 2020 [68]	Uzbekistan (healthcare facility)	“Patient and health-care worker perspectives on the short-course regimen for treatment of drug-resistant tuberculosis in Karakalpakstan, Uzbekistan”	44	Patients and healthcare workers	Qualitative
50	Horter et al., 2016 [69]	Uzbekistan (healthcare facility)	“Where there is hope: a qualitative study examining patients’ adherence to multi-drug resistant tuberculosis treatment in Karakalpakstan, Uzbekistan”	52	Patients and healthcare workers	Qualitative
51	Hosoglu et al., 2021 [70]	Turkey (medical center and outpatient clinic)	“Antibiotic prescription in primary care from the perspective of family physicians: a qualitative study”	14	Family physicians	Qualitative
52	Hoxha et al., 2018 [71]	Albania (pharmacies)	“Are pharmacists’ good knowledge and awareness on antibiotics taken for granted? The situation in Albania future implications across countries”	370	Community pharmacists	Qualitative
53	Huong et al., 2021 [72]	Vietnam (acute-care hospitals)	“Improving antimicrobial use through antimicrobial stewardship in a lower-middle income setting: a mixed-methods study in a network of acute-care hospitals in Vietnam”	40	Hospital staff on AMR and AMS programs	Mixed methods
54	Inchara et al., 2022 [73]	India (rural tertiary care center)	“‘Perceptions’ and ‘practices’ to antibiotic usage among diabetic patients receiving care from a rural tertiary care center: A mixed-methods study”	5	Patients	Mixed methods
55	Intahphuak et al., 2022 [74]	Thailand (community primary care centers)	“Community Health Nurses’ Perspective on the Introduced Rational Drug Use Policy in Primary Care Settings in Thailand: A Descriptive Qualitative Study”	12	Community health nurses	Qualitative
56	Jakupi et al., 2019 [75]	Kosovo (health facilities, pharmacies and cafes)	“Culture of antibiotic use in Kosovo—an interview study with patients and health professionals”	16	Patients, community pharmacists and physicians	Qualitative
57	Kaae et al., 2020 [76]	Russia federation (hospitals, medical universities and polyclinics)	“The antibiotic knowledge, attitudes and behaviors of patients, doctors and pharmacists in the WHO Eastern European region—a qualitative, comparative analysis of the culture of antibiotic use in Armenia, Georgia, Kazakhstan, Moldova, Russia and Tajikistan”	80	Patients, doctors and pharmacists	Qualitative
58	Kaae et al., 2017 [77]	Albania (hospitals and pharmacies)	“Antibiotic knowledge, attitudes and behaviors of Albanian healthcare professionals and patients—a qualitative interview study”	16	Patients, community pharmacists, and physicians	Qualitative
59	Kagoya et al., 2021 [78]	Uganda (regional referral hospitals)	“Experiences and views of healthcare professionals on the prescription of antibiotics in Eastern Uganda: A qualitative study”	16	Doctors, nurses and clinical officers	Mixed methods
60	Kandeel et al., 2014 [79]	Egypt (pharmacies, healthcare facilities, and at the community level)	“Patient Attitudes and Beliefs and Provider Practices Regarding Antibiotic Use for Acute Respiratory Tract Infections in Minya, Egypt”	160	Healthcare providers and patients	Mixed methods
61	Khan et al., 2021 [80]	Pakistan (community pharmacies)	“Knowledge, Attitude, and Practice on Antibiotics and Its Resistance: A Two-Phase Mixed-Methods Online Study among Pakistani Community Pharmacists to Promote Rational Antibiotic Use”	21	Community pharmacists	Mixed methods
62	Khan et al., 2022 [81]	Pakistan (community pharmacies)	“Evaluation of Consumers Perspective on the Consumption of Antibiotics, Antibiotic Resistance, and Recommendations to Improve the Rational use of Antibiotics: An Exploratory Qualitative Study From Post-Conflicted Region of Pakistan”	20	Clients of pharmacies	Qualitative
63	Khan et al., 2021 [82]	India (medical college hospital)	“Qualitative Thematic Analysis of Knowledge and Practices of Surgical Antimicrobial Prophylaxis at a Tertiary Care Teaching Hospital”	184	Medical consultants and surgeons	Mixed methods
64	Khan et al., 2020 [83]	Pakistan and Cambodia	“Is enhancing the professionalism of healthcare providers critical to tackling antimicrobial resistance in low- and middle-income countries?”	85	Healthcare providers, policymakers, and pharmaceutical industry representatives	Qualitative
65	Khare et al., 2022 [84]	India (rural areas in Ujjain District, Madhya Pradesh)	“Understanding Internal and External Drivers Influencing the Prescribing Behaviour of Informal Healthcare Providers with Emphasis on Antibiotics in Rural India”	48	Informal healthcare providers	Qualitative
66	Kotwani and Gandra, 2023 [85]	India (secondary and primary public healthcare facilities)	“Strengthening antimicrobial stewardship activities in secondary and primary public healthcare facilities in India”	Not indicated	Doctors, public health special specialists, health directors	Qualitative
67	Kotwani et al., 2021 [86]	India (pharmacies)	“Over-the-Counter Sale of Antibiotics in India: A Qualitative Study of Providers’ Perspectives across Two States”	22	Pharmacists and informal dispensers	Qualitative
68	Kpokiri et al., 2020 [87]	Nigeria (hospitals)	“Development of Antimicrobial Stewardship Programmes in Low and Middle-Income Countries: A Mixed-Methods Study in Nigerian Hospitals”	17	Physicians	Mixed methods
69	Kuijpers et al., 2018 [88]	Cambodia (hospitals, pharmacies, and at the community level)	“Enteric Fever in Cambodia: Community Perceptions and Practices Concerning Disease Transmission and Treatment”	39	Patients	Qualitative
70	Kukula et al., 2023 [89]	Ghana (public health facilities)	“Understanding Health Worker and Community Antibiotic Prescription-Adherence Practices for Acute Febrile Illness: A Nested Qualitative Study in the Shai-Osudoku District of Ghana and the Development of a Training-and-Communication Intervention”	105	Health workers and community members	Qualitative
71	Lai et al., 2022 [90]	Malaysia (public hospitals)	“Pharmacists’ Perspectives of Their Roles in Antimicrobial Stewardship: A Qualitative Study among Hospital Pharmacists in Malaysia”	16	Hospital pharmacists	Qualitative
72	Legba et al., 2023 [91]	Benin (laboratories, hospitals, pharmacies, unattached maternity wards)	“Assessment of blood cultures and antibiotic susceptibility testing for bacterial sepsis diagnosis and utilization of results by clinicians in Benin: A qualitative study”	159	Laboratory staff, physicians and pharmacists	Qualitative
73	Legenza et al., 2018 [92]	South Africa (secondary hospitals)	“Clostridium difficile infection perceptions and practices: a multicenter qualitative study in South Africa”	36	Physicians, nurses, pharmacists	Qualitative
74	Limato et al., 2022 [93]	Indonesia (hospitals)	“A qualitative study of barriers to antimicrobial stewardship in Indonesian hospitals: governance, competing interests, cost, and structural vulnerability”	51	Physicians, surgeons, clinical microbiologists, clinical pharmacists, hospital AMS team leaders, hospital managers, medical students, and national AMR stakeholders	Qualitative
75	Maki et al., 2020 [94]	Bhutan (healthcare facilities)	“Feasibility Study of the World Health Organization Health Care Facility-Based Antimicrobial Stewardship Toolkit for Low- and Middle-Income Countries”	98	Administrators, physicians, nurses, pharmacists, and laboratory personnel	Qualitative
76	Malazarte et al., 2024 [95]	Philippines (hospitals)	“Hospital pharmacists’ expertise and cooperation towards antimicrobial stewardship in the Philippines: A qualitative study”	19	Hospital pharmacists	Qualitative
77	Manderson, 2020 [96]	South Africa (clinics, private medical centers and government health facilities)	“Prescribing, care and resistance: antibiotic use in urban South Africa”	102	Doctors, nurses, pharmacists, patients, advocates of antibiotic guardianship	Qualitative
78	Mao et al., 2023 [97]	Cambodia (hospital and national reference laboratory)	“The barriers and facilitators of implementing a national laboratory-based AMR surveillance system in Cambodia: key informants’ perspectives and assessments of microbiology laboratories”	25	Surveillance personnel and high-level management	Mixed methods
79	Marasini et al., 2024 [98]	Nepal (community pharmacies and health outreach centers)	“Exploring knowledge, perceptions, and practices of antimicrobials, and their resistance among medicine dispensers and community members in Kavrepalanchok District of Nepal”	25	Medicine dispensers and community members	Qualitative
80	Mathew et al., 2020 [99]	India (Hospitals)	“Challenges in Implementing Antimicrobial Stewardship Programmes at Secondary Level Hospitals in India: An Exploratory Study”	5	Doctors, pharmacologists, pharmacists and intensivists	Qualitative
81	Matin et al., 2020 [100]	Bangladesh (Public and private drug outlets)	“What influences antibiotic sales in rural Bangladesh? A drug dispensers’ perspective”	16	Drug dispensers	Mixed methods
82	Mattingly et al., 2019 [101]	Ethiopia (Hospitals)	“Qualitative outcomes of Clean Cut: implementation lessons from reducing surgical infections in Ethiopia”	20	Surgeons, anesthetists, operating room nurses, operating room ward nurses, operating room managers, quality improvement personnel, and hospital administrators	Qualitative
83	Mbugua et al., 2020 [102]	Kenya (Referral hospitals)	“Exploring perspectives on antimicrobial stewardship: a qualitative study of health managers in Kenya”	8	Medical superintendents, nurse managers, hospital pharmacists and pharmacist manager	Qualitative
84	McKnight et al., 2020 [103]	Kenya (Public hospitals)	“Evaluating hospital performance in antibiotic stewardship to guide action at national and local levels in a lower-middle income setting”	31	Hospital managers, frontline HCWs including consultants, medical officers, nursing officers, pharmacists, and laboratory technicians	Mixed methods
85	Mmari et al., 2021 [104]	Tanzania (Tertiary hospital)	“Perceptions of surgeons on surgical antibiotic prophylaxis use at an urban tertiary hospital in Tanzania”	14	Surgeons, obstetrician and gynecologists	Qualitative
86	Mohr et al., 2018 [105]	South Africa (Primary healthcare facilities)	““Life continues”: Patient, health care and community care workers perspectives on self-administered treatment for rifampicin-resistant tuberculosis in Khayelitsha, South Africa”	9	Patients	Concurrent mixed-method study
87	Mula et al., 2019 [106]	Malawi (Tertiary hospital)	“An exploration of workarounds and their perceived impact on antibiotic stewardship in the adult medical wards of a referral hospital in Malawi: a qualitative study”	33	Medical doctors, pharmacists, laboratory technologists and nurses	Qualitative
88	Mula et al., 2021 [107]	Malawi (Referral hospital)	“Nurses’ role in antibiotic stewardship at medical wards of a referral hospital in Malawi: Understanding reality and identifying barriers”	43	Pharmacists, laboratory technologists, medical doctors and nurses	Qualitative
89	Mula et al., 2019 [108]	Malawi (Tertiary hospital)	“The examination of nurses’ adherence to the ‘five rights’ of antibiotic administration and factors influencing their practices: a mixed methods case study at a tertiary hospital, Malawi”	13	Nurses	Mixed methods
90	Mussie et al., 2019 [109]	Ethiopia (Public healthcare facilities)	“Exploring local realities: Perceptions and experiences of healthcare workers on the management and control of drug-resistant tuberculosis in Addis Ababa, Ethiopia”	18	Clinical nurses, health officers, and medical laboratory technicians	Qualitative
91	Nair et al., 2019 [110]	India (Primary health centers and hospitals)	““Without antibiotics, I cannot treat”: A qualitative study of antibiotic use in Paschim Bardhaman district of West Bengal, India”	28	Allopathic doctors, informal health providers, nurses, pharmacy shopkeepers, patients	Qualitative
92	Nepal et al., 2020 [111]	Nepal (Public and private health facilities)	“Factors influencing the inappropriate use of antibiotics in the Rupandehi district of Nepal”	17	Physicians, health workers, dispensers, district policymakers	Qualitative
93	Nguyen et al., 2019 [112]	Vietnam (Public hospital pharmacy, community health center, private dispensaries traditional medicine center, private clinics)	““I can make more from selling medicine when breaking the rules”—understanding the antibiotic supply network in a rural community in Vietnam”	81	Drug suppliers	Qualitative
94	Niaz et al., 2020 [113]	Namibia (Public healthcare facilities)	“Compliance to prescribing guidelines among public health care facilities in Namibia; findings and implications”	37	Prescribers	Mixed methods
95	Nokhodian et al., 2024 [114]	Iran (Hospitals and communities setting)	“Overuse of Antibiotics: Who is to Blame? A Qualitative Study”	13	Clinicians	Qualitative
96	Okwera et al., 2015 [115]	Uganda (national referral tuberculosis treatment center and national referral and teaching hospital)	“Level of understanding of co-trimoxazole use among HIV infected, recurrent pulmonary tuberculosis suspects at a national referral tuberculosis clinic in Kampala, Uganda: a qualitative analysis”	30	Patients	Qualitative
97	Om et al., 2016 [116]	Cambodia (public hospitals)	““If it’s a broad spectrum, it can shoot better”: inappropriate antibiotic prescribing in Cambodia”	103	Physicians	Qualitative
98	Rachina et al., 2023 [117]	Russia (social networks, pharmacies, polyclinics, and hospitals)	“The Antibiotic Knowledge, Attitudes, and Behaviors of Patients Purchasing Antibiotics without Prescription: Results of National Survey”	149	Patients	Qualitative
99	Ravi et al., 2017 [118]	India (oncology hospital and center)	“Exploring the Prescribing Behaviours and the Mind of Antibiotic Prescribers is Critical for a Successful Antibiotic Stewardship Programme: Results of a Survey from Eastern India”	5	Consultants	Mixed methods
100	Raza et al., 2024 [119]	Pakistan (public and private hospitals)	“Awareness amongst service providers and patients regarding the use and dispense of antibiotics a cross-city analysis in Pakistan”	14	Healthcare providers and patients	Qualitative
101	Rolfe et al., 2021 [120]	Sri Lanka, Kenya, Tanzania (tertiary care hospitals)	“Barriers to implementing antimicrobial stewardship programs in three low- and middle-income country tertiary care settings: findings from a multi-site qualitative study”	45	Physicians	Qualitative
102	Rout, 2015 [121]	South Africa (20-bed ICU, private hospital, KwaZulu-Natal)	“Exploring the role of the ICU nurse in the antimicrobial stewardship team at a private hospital in KwaZulu-Natal, South Africa”	15	ICU nurses, nursing managers, anesthetists, physicians, surgeons), microbiologist, pharmacist	Qualitative
103	Rout and Brysiewicz, 2020 [122]	South Africa (private hospital)	“Perceived barriers to the development of the antimicrobial stewardship role of the nurse in intensive care: Views of healthcare professionals”	16	ICU nurses, nursing managers, anesthetists, physicians, surgeons), microbiologist, pharmacist	Qualitative
104	Royce et al., 2014 [123]	Cambodia (Ministry of Health, partner organizations, referral hospital, and health centers	“Identification of multidrug resistance in previously treated tuberculosis patients: a mixed methods study in Cambodia”	26	Doctors or clinical officers, nurses, laboratory staff, and TB officers	Mixed method
105	Saleem et al., 2019 [124]	Pakistan (office, residence, clinic, hospital)	“Antimicrobial prescribing and determinants of antimicrobial resistance: a qualitative study among physicians in Pakistan”	15	Physicians	Qualitative
106	Saleem et al., 2019 [125]	Pakistan (office, residence, clinic, hospital)	“Antimicrobial dispensing practices and determinants of antimicrobial resistance: a qualitative study among community pharmacists in Pakistan”	12	Community pharmacists	Qualitative
107	Saleh et al., 2021 [126]	Jordan (community pharmacies)	“Views of Community Pharmacists on Antimicrobial Resistance and Antimicrobial Stewardship in Jordan: A Qualitative Study”	20	Community pharmacists	Qualitative
108	Salem et al., 2023 [127]	Egypt (university hospitals)	“Perspectives on Antibiotic Stewardship Programs among Health Care Providers at Two University Hospitals in Egypt”	43	Physicians and pharmacists	Qualitative
109	Salim and Elgizoli, 2017 [128]	Sudan (community pharmacies)	“Exploring the reasons why pharmacists dispense antibiotics without prescriptions in Khartoum state, Sudan”	30	Community pharmacists	Qualitative
110	Sami et al., 2022 [129]	Iran (university hospital)	“Barriers to rational antibiotic prescription in Iran: a descriptive qualitative study”	36	Physicians	Qualitative
111	Sneddon et al., 2022 [130]	Ghana (hospitals)	“Exploring the Use of Antibiotics for Dental Patients in a Middle-Income Country: Interviews with Clinicians in Two Ghanaian Hospitals”	12	Dentists, pharmacists, pharmacy technicians, dental surgery assistant, physician assistant	Qualitative
112	Stringer et al., 2016 [131]	Uzbekistan (health facility)	“‘They prefer hidden treatment’: anti-tuberculosis drug-taking practices and drug regulation in Karakalpakstan”	24	Patients and practitioners	Qualitative
113	Sultana et al., 2023 [132]	Bangladesh (healthcare-providing centers)	“Physicians’ Antibiotics Prescribing Patterns for Common Diseases and Knowledge on Antimicrobial Resistance: A Descriptive Cross-Sectional Study”	33	Patients and caregivers, allied health personnel, health policy makes	Mixed methods
114	Torres et al., 2019 [133]	Mozambique (pharmacies)	“Patterns of self-medication with antibiotics in Maputo City: a qualitative study”	49	Pharmacy customers and pharmacists	Qualitative
115	Torres et al., 2023 [134]	Mozambique (pharmacies)	““Antibiotics heal all diseases”; the factors influencing the practices of self-medication with antibiotics in Maputo City, Mozambique”	32	Community and pharmacy customers	Qualitative
116	Torres et al., 2020 [135]	Mozambique (pharmacies)	“Pharmacists’ practices for non-prescribed antibiotic dispensing in Mozambique”	17	Pharmacists	Qualitative
117	Ubiztondo et al., 2018 [136]	Argentina, Bolivia, Paraguay, Uruguay (primary healthcare centers)	“General Practitioners’ Views on the Acceptability and Applicability of Using Quality Indicators as an Intervention to Reduce Unnecessary Prescription of Antibiotics in Four South American Countries”	82	General practitioners	Qualitative
118	van Gulik et al., 2021 [137]	Thailand (university public hospital)	“Perceived roles and barriers to nurses’ engagement in antimicrobial stewardship: A Thai qualitative case study”	15	Directors, managers, physicians and nurses	Qualitative
119	Van Hecke et al., 2019 [138]	South Africa (primary care clinics)	“Introducing new point-of-care tests for common infections in publicly funded clinics in South Africa: a qualitative study with primary care clinicians”	23	Nurses and doctors	Qualitative

**Table 2:** Quality assessment of studies included in the review using the CASP tool.

No.	Author, Year	Aim Clarity	Method Appropriateness	Research Design	Sampling Design/Recruitment strategy	Data Collection	Reflexivity	Ethical Issues	Data Analysis	Findings	Research Value	Score (/10)
1	Abdelaziz et al., 2019 [21]	1	0	1	0.5	1	0	1	0.5	1	1	7
2	Abdel Jalil et al., 2023 [22]	1	1	1	0.5	1	0.5	1	0.5	1	1	8.5
3	Abu Mhadi et al., 2023 [23]	1	1	1	0.5	1	0.5	1	0.5	1	1	8.5
4	Ackers et al., 2020 [24]	1	1	1	1	1	0.5	1	0.5	1	1	9
5	Afari-Asiedu et al., 2021 [25]	1	1	1	0.5	1	0	1	0.5	1	1	8
6	Aika and Enato, 2022 [26]	1	1	1	0.5	1	0.5	1	0.5	1	1	8.5
7	Akhtar et al., 2020 [27]	1	0.5	1	0.5	1	0.5	1	0.5	1	1	8
8	Alemkere et al., 2023 [28]	1	1	1	1	1	0.5	1	1	1	1	9.5
9	Älgå et al., 2018 [29]	1	1	1	1	1	0.5	1	1	1	1	9.5
10	Älgå et al., 2018 [29]	1	1	1	1	1	0.5	1	0.5	1	1	9
11	Alghamdi et al., 2021 [30]	1	1	1	0.5	1	0	1	0.5	1	1	8
12	Alkadhimi et al., 2021 [31]	1	0.5	1	0.5	1	0.5	1	0.5	0.5	1	7.5
13	Al Meslamani et al., 2022 [32]	1	1	1	1	1	0.5	1	1	1	1	9.5
14	Amin et al., 2017 [33]	1	1	1	0.5	1	0.5	1	1	1	1	9
15	Anwar et al., 2021 [34]	1	1	1	1	1	0.5	1	1	1	1	9.5
16	Asante et al., 2017 [35]	1	1	1	0.5	1	0.5	1	1	1	1	9
17	Atif et al., 2020 [36]	1	1	1	0.5	1	0.5	1	1	1	1	9
18	Atif et al., 2019 [37]	1	1	1	1	1	0.5	1	1	1	1	9.5
19	Atif et al., 2021 [38]	1	1	1	0.5	0.5	0	1	0.5	1	0.5	7
20	Bahnassi [39]	1	1	1	1	1	0.5	1	1	1	1	9.5
21	Bahta et al., 2021 [40]	1	1	1	0.5	1	0.5	1	1	1	1	9
22	Bajwa et al., 2024 [41]	1	1	1	0.5	1	0.5	1	1	1	1	9
23	Barker et al., 2017 [42]	1	1	1	0.5	1	0	1	0.5	1	1	8
24	Baubie et al., 2019 [43]	1	1	1	1	1	0.5	1	1	1	1	9.5
25	Belachew et al., 2023 [44]	1	1	1	0.5	1	0.5	1	1	1	1	9
26	Broom and Doron, 2020 [45]	1	1	1	0.5	1	0.5	1	1	1	1	9
27	Broom et al., 2021 [46]	1	1	0.5	0.5	0.5	0.5	1	0.5	1	1	7.5
28	Cantarero-Arevalo et al., 2022 [47]	1	1	1	1	1	0.5	1	1	1	1	9.5
29	Chansamouth et al., 2024 [48]	1	1	1	1	1	1	1	1	1	1	10
30	Chukwu et al., 2024 [49]	1	1	1	0.5	1	0.5	1	0.5	1	1	8.5
31	Darj et al., 2020 [50]	1	1	1	0.5	1	0.5	1	1	1	1	9
32	Di et al., 2022 [51]	1	1	1	0.5	1	0.5	1	0.5	1	1	8.5
33	Dillip et al., 2015 [52]	1	1	1	1	1	0.5	1	0.5	1	1	9
34	Dohou et al., 2022 [53]	1	1	1	0.5	1	0.5	0.5	1	1	1	8.5
35	Dooling et al., 2014 [54]	1	1	1	1	1	0.5	1	0.5	1	1	9
36	Do et al., 2021 [55]	1	1	1	1	1	0.5	1	0.5	1	1	9
37	Edessa et al., 2024 [56]	1	1	1	1	1	0.5	1	0.5	1	1	9
38	Eibs et al., 2020 [57]	1	1	1	1	0.5	0.5	1	0.5	1	1	8.5
39	Esfandiari et al., 2018 [58]	1	1	1	1	1	0.5	1	1	1	1	9.5
40	Farooqui et al., 2023 [59]	1	1	1	1	1	0.5	1	1	1	1	9.5
41	Foster and Bandawe, 2014 [60]	1	0.5	0.5	0.5	0.5	0	1	0	0.5	1	5.5
42	Gautham et al., 2021 [61]	1	1	1	1	1	0.5	1	1	1	1	9.5
43	Gebretekle et al., 2018 [62]	1	1	1	0.5	1	0.5	1	0.5	1	1	8.5
44	Gebretekle and Serbessa, 2016 [63]	1	1	1	0.5	1	0.5	0.5	1	1	1	8.5
45	Ghiga et al., 2023 [64]	1	1	1	0.5	1	0.5	1	1	1	1	9
46	Ghiga and Lundborg, 2016 [65]	1	1	1	0.5	1	0.5	1	1	1	1	9
47	Green et al., 2023 [66]	1	1	1	0.5	1	0.5	1	1	1	1	9
48	Hayat et al., 2019 [67]	1	1	1	1	1	0.5	1	1	1	1	9.5
49	Horter et al., 2020 [68]	1	1	1	1	1	0.5	1	1	1	1	9.5
50	Horter et al., 2016 [69]	1	1	1	0.5	1	0.5	1	1	1	1	9
51	Hosoglu et al., 2021 [70]	1	1	1	0.5	1	0.5	1	0.5	1	1	8.5
52	Hoxha et al., 2018 [71]	1	0.5	1	1	1	0.5	0.5	0.5	1	1	8.5
55	Huong et al., 2021 [72]	1	1	1	0.5	1	0.5	1	1	1	1	9
54	Inchara et al., 2022 [73]	1	1	1	1	1	0.5	1	1	1	1	9.5
55	Intahphuak et al., 2022 [74]	1	1	1	0.5	1	0.5	1	0.5	1	1	8.5
56	Jakupi et al., 2019 [75]	1	1	1	0.5	0.5	0.5	1	0.5	1	1	8
57	Kaae et al., 2020 [76]	1	1	1	0.5	1	0.5	1	1	1	1	9
58	Kaae et al., 2017 [77]	1	1	1	0.5	0.5	0.5	0.5	1	1	1	8
59	Kagoya et al., 2021 [78]	1	1	1	0.5	1	0.5	1	1	1	1	9
60	Kandeel et al., 2014 [79]	1	1	1	0.5	0.5	0	1	0.5	1	1	7.5
61	Khan et al., 2021 [80]	1	1	1	0.5	1	0	1	0.5	1	1	8
62	Khan et al., 2022 [81]	1	1	1	0.5	1	0.5	1	0.5	1	1	8.5
63	Khan et al., 2021 [82]	1	1	1	1	1	0	0.5	1	1	1	8.5
64	Khan et al., 2020 [83]	1	1	1	1	1	0.5	1	1	1	1	9.5
65	Khare et al., 2022 [84]	1	1	1	0.5	1	0.5	1	1	1	1	9
66	Kotwani and Gandra, 2023 [85]	1	1	1	0.5	0.5	0	0.5	0.5	1	1	7
67	Kotwani et al., 2021 [86]	1	1	1	0.5	1	0.5	1	1	1	1	9
68	Kpokiri et al., 2020 [87]	1	1	1	1	1	0.5	1	0.5	1	1	9
69	Kuijpers et al., 2018 [88]	1	1	1	1	1	0.5	1	1	1	1	9.5
70	Kukula et al., 2023 [89]	1	1	1	1	1	0.5	1	1	1	1	9.5
71	Lai et al., 2022 [90]	1	1	1	1	1	0.5	1	1	1	1	9.5
72	Legba et al., 2023 [91]	1	1	0.5	1	1	0.5	0.5	0.5	0.5	1	7.5
73	Legenza et al., 2018 [92]	1	1	1	0.5	1	0.5	1	1	1	1	9
74	Limato et al., 2022 [93]	1	1	1	1	1	0.5	1	1	1	1	9.5
75	Maki et al., 2020 [94]	1	1	1	1	1	0.5	1	0.5	1	1	9
76	Malazarte et al., 2024 [95]	1	1	1	0.5	1	0.5	1	1	1	1	9
77	Manderson, 2020 [96]	1	1	1	1	1	0.5	1	1	1	1	9.5
78	Mao et al., 2023 [97]	1	1	1	1	1	0.5	1	1	1	1	9.5
79	Marasini et al., 2024 [98]	1	1	1	1	1	0.5	1	1	1	1	9.5
80	Mathew et al., 2020 [99]	1	1	1	0.5	1	0.5	1	0.5	1	1	8.5
81	Matin et al., 2020 [100]	1	1	1	1	1	0.5	1	1	1	1	9.5
82	Mattingly et al., 2019 [101]	1	1	1	1	1	0.5	1	1	1	1	9.5
83	Mbugua et al., 2020 [102]	1	1	1	0.5	1	0.5	1	1	1	1	9
84	McKnight et al., 2020 [103]	1	1	1	1	1	0.5	1	1	1	1	9.5
85	Mmari et al., 2021 [104]	1	1	1	0.5	1	0.5	1	1	1	1	9
86	Mohr et al., 2018 [105]	1	1	1	0.5	1	1	1	0.5	1	1	9
87	Mula et al., 2019 [106]	1	1	1	1	1	0.5	1	1	1	1	9.5
88	Mula et al., 2021 [107]	1	1	1	1	1	1	1	1	1	1	10
89	Mula et al., 2019 [108]	1	1	1	1	1	0.5	1	1	1	1	9.5
90	Mussie et al., 2019 [109]	1	1	1	0.5	1	0.5	1	1	1	1	9
91	Nair et al., 2019 [110]	1	1	1	0.5	1	0.5	1	0.5	1	1	8.5
92	Nepal et al., 2020 [111]	1	1	1	1	1	0.5	1	1	1	1	9.5
93	Nguyen et al., 2019 [112]	1	1	1	0.5	1	0.5	1	1	1	1	9
94	Niaz et al., 2020 [113]	1	1	1	1	1	0.5	1	1	1	1	9.5
95	Nokhodian et al., 2024 [114]	1	1	1	0.5	1	0.5	1	0.5	1	1	8.5
96	Okwera et al., 2015 [115]	1	1	1	1	1	0.5	1	1	1	1	9.5
97	Om et al., 2016 [116]	1	1	1	0.5	1	0	1	0.5	1	1	8
98	Rachina et al., 2023 [117]	1	1	1	1	1	0.5	1	1	1	1	9.5
99	Ravi et al., 2017 [118]	1	1	1	0.5	1	0.5	1	0.5	1	1	8.5
100	Raza et al., 2024 [119]	1	1	0.5	0.5	1	0	0.5	0.5	1	1	7
101	Rolfe et al., 2021 [120]	1	1	1	0.5	1	0	0.5	1	1	1	8
102	Rout, 2015 [121]	1	1	1	0.5	1	0.5	1	1	1	1	9
103	Rout and Brysiewicz, 2020 [122]	1	1	1	1	0.5	1	1	1	1	1	9.5
104	Royce et al., 2014 [123]	1	1	1	0.5	1	0.5	1	1	1	1	9
105	Saleem et al., 2019 [124]	1	1	1	1	1	0.5	1	1	1	1	9.5
106	Saleem et al., 2019 [125]	1	1	1	1	1	0.5	1	1	1	1	9.5
107	Saleh et al., 2021 [126]	1	1	1	0.5	1	0.5	1	0.5	1	1	8.5
108	Salem et al., 2023 [127]	1	1	0.5	0.5	0.5	0.5	1	0.5	0.5	0.5	6.5
109	Salim and Elgizoli, 2017 [128]	1	1	1	0.5	1	0.5	1	0.5	1	1	8.5
110	Sami et al., 2022 [129]	1	1	1	0.5	1	0.5	1	0.5	1	1	8.5
111	Sneddon et al., 2022 [130]	1	1	1	1	1	0.5	1	1	1	1	9.5
112	Stringer et al., 2016 [131]	1	1	1	0.5	1	0.5	1	1	1	1	9
113	Sultana et al., 2023 [132]	1	1	1	1	1	0.5	1	1	1	1	9.5
114	Torres et al., 2019 [133]	1	1	1	1	1	0.5	1	1	1	1	9.5
115	Torres et al., 2023 [134]	1	1	0.5	0.5	1	0.5	1	0.5	1	1	8
116	Torres et al., 2020 [135]	1	1	1	1	1	1	1	1	1	1	10
117	Ubiztondo et al., 2018 [136]	1	1	1	1	1	0.5	1	0.5	1	1	9
118	van Gulik et al., 2021 [137]	1	1	1	1	1	0.5	1	0.5	1	1	9
119	Van Hecke et al., 2019 [138]	1	1	1	0.5	1	1	1	1	1	1	9.5

**Table 3:** Final themes, sub-themes, codes and supporting studies.

Theme	Key Sub-Themes	Supporting Codes from Data	Studies from Dataset
Financial, Commercial, Socioeconomic, and Cultural Drivers of Misuse	Poverty-driven self-medication; patient pressure; informal markets; cultural trust in antibiotics	Patient demand, financial incentives, poverty, self-medication, cultural beliefs, trust in antibiotics	Atif et al., 2019 [37]; Green et al., 2023 [66]; Nguyen et al., 2019 [112]; Gautham et al., 2021 [61]
The Disconnect Between Knowledge, Sociocultural Norms and Practice	Knowledge gaps; guideline non-adherence; misconceptions about resistance; “strong medicine” belief	Knowledge gaps, misinterpretation of resistance, guidelines not followed, antibiotics seen as “strong medicine”	Älgå et al., 2018 [29]; Om et al., 2016 [116]; Khan et al., 2021 [80]; Kaae et al., 2020 [76]
Antibiotic Use as a Pragmatic Response to Systemic and Structural Constraints	Weak regulation; lack of diagnostics; resource constraints; commercial influences	Weak regulation enforcement, lack of diagnostic tools, resource limitations, pharmaceutical influence	Limato et al., 2022 [93]; Legba et al., 2023 [91]; Rolfe et al., 2021 [120]; Kotwani & Gandra, 2023 [85]
Fragmented Stewardship: Organizational Gaps, Role Ambiguity, and Professional Tensions	Role ambiguity; underutilized cadres; inter-professional conflicts; lack of training	Role ambiguity, nurses’ underutilized role, prescriber–pharmacist conflicts, lack of stewardship training	Mula et al., 2021 [107]; van Gulik et al., 2021 [137]; Rout, 2015 [121]; Aika & Enato, 2022 [26]
Informal and Market-Driven Antibiotic Use Beyond Formal Stewardship Systems	Informal sector dominance; OTC norms; parallel supply chains; profit-driven dispensing	Over-the-counter sale norms, informal provider practices, financial incentives, weak regulation enforcement	Alkadhimi et al., 2021 [31]; Barker et al., 2017 [42]; Kotwani et al., 2021 [86]; Bahta et al., 2021 [40]

## Data Availability

The raw data supporting the conclusions of this article will be made available by the authors on request.

## Supplementary Materials

The following supporting information can be downloaded at: https://www.mdpi.com/article/10.3390/antibiotics15050468/s1, Documentation of search strategies, PRISMA 2020 Checklist, PRISMA 2020 Abstract Checklist.
