# Supplementary material for: Perspectives and Challenges of Healthcare Professionals, Patients, and Caregivers Regarding Utilizing Antibiotics and Implementing Antibiotic Stewardship in Healthcare Facilities in Low- and Middle-Income Countries: A Systematic Review of Qualitative Studies
Source: Antibiotics (Basel). 2026 May 5;15(5):468. doi: 10.3390/antibiotics15050468 (PMC13203857; doi:10.3390/antibiotics15050468)
Supplement: Supplementary file 1 [file antibiotics-15-00468-s001.zip › 260325 Documentation of search strategies_ Systematic review_Bode Ireti Shobayo.pdf]

Karolinska Institutet

Documentation of search strategies
University Library search request group

Topic/research question: Perspectives and challenges of healthcare professionals, patients, and caregivers regarding utilizing antibiotics and implementing Antibiotic stewardship in healthcare facilities in LMICs; a systematic review of qualitative studies

Name of researcher(s): Bode Ireti Shobayo, Global public health

Librarian(s): Emma-Lotta Säätelä & AnnaMia Eborn Martinovic

Databases:

1. Medline (Ovid)
2. Embase (embase.com)
3. Cochrane Library (Wiley)
4. Web of Science (Clarivate Analytics)

Complementary search in:

5. Google scholar

Karolinska Institutet

1. Medline

Interface: **Ovid MEDLINE(R) ALL** content coverage from 1946<br><br>Date of Search: 15 October 2024<br><br>Number of hits: 979<br><br>Comment: In Ovid, two or more words are automatically searched as phrases; i.e. no quotation marks are needed	Field labels<br><br>• exp/ = exploded MeSH term<br>• / = non exploded MeSH term<br>• .ti,ab,kf. = title, abstract and author keywords<br>• adjx = within x words, regardless of order<br>• * = truncation of word for alternate endings<br>• ? = 0-1 letter/number<br>• # = 1 letter/number

Database(s): **Ovid MEDLINE(R) ALL** 1946 to October 10, 2024
Search Strategy:

#	Searches	Results
1	exp "Interviews as topic"/	69828
2	exp Focus groups/	38741
3	exp Narration/	11040
4	exp Qualitative research/	93957
5	exp "Personal narratives as topic"/	359
6	(("semi-structured" or semistructured or unstructured or informal or "in-depth" or indepth or "face-to-face" or structured or guide*) adj3 (interview* or discussion* or questionnaire*)).ti,ab,kf.	204585
7	(focus group* or qualitative or ethnograph* or fieldwork or "field work" or "key informant").ti,ab,kf.	418867
8	((mixed or multiple) adj4 (design or method* or study)).ti,ab,kf.	129308
9	(multimethod* or multi-method*).ti,ab,kf.	5935
10	or/1-9	675838
11	exp Anti-Bacterial Agents/	849202
12	Antimicrobial Stewardship/	3879
13	exp Drug resistance, microbial/	189335
14	(antibacterial or anti-bacterial or antimicrobial or anti-microbial or antibiotic* or anti-biotic*).ti,ab,kf.	702199
15	or/11-14	1258198

16	Health Knowledge, Attitudes, Practice/	131903
17	Attitude/	55088
18	Attitude of health personnel/	135310
19	Attitude to health/	85507
20	Emotions/	91530
21	(experience or experiences or perspective? or perception? or emotion? or feelings or attitude? or view?).ti,ab,kf.	2429544
22	or/16-21	2652449
23	Medically Underserved Area/ or Developing Countries/ or Rural Health/ or Rural Population/	178140
24	Afghanistan/ or Albania/ or Algeria/ or Angola/ or Argentina/ or Armenia/ or Azerbaijan/ or Bangladesh/ or Benin/ or "Republic of Belarus"/ or Belize/ or Bhutan/ or Bolivia/ or "Bosnia and Herzegovina"/ or Botswana/ or Brazil/ or Bulgaria/ or Burkina Faso/ or Burundi/ or Cambodia/ or Cameroon/ or Cabo Verde/ or Central African Republic/ or Chad/ or Colombia/ or Comoros/ or Congo/ or Costa Rica/ or Cote d'Ivoire/ or Cuba/ or Djibouti/ or Dominican Republic/ or Ecuador/ or Egypt/ or El Salvador/ or Eritrea/ or Equatorial Guinea/ or Ethiopia/ or Fiji/ or Gabon/ or Gambia/ or Georgia/ or Ghana/ or Grenada/ or Guatemala/ or Guinea/ or Guinea-Bissau/ or Guyana/ or Haiti/ or Honduras/ or India/ or Indonesia/ or Iran/ or Iraq/ or Jamaica/ or Jordan/ or Kazakhstan/ or Kenya/ or "Democratic People's Republic of Korea"/ or Kosovo/ or Kyrgyzstan/ or Laos/ or Lebanon/ or Lesotho/ or Liberia/ or Libya/ or Macedonia/ or Madagascar/ or Malaysia/ or Malawi/ or Mali/ or Mauritania/ or Micronesia/ or Mauritius/ or Mexico/ or Moldova/ or Mongolia/ or Montenegro/ or Morocco/ or Mozambique/ or Myanmar/ or Namibia/ or Nepal/ or Nicaragua/ or Niger/ or Nigeria/ or Pakistan/ or Palau/ or "Papua New Guinea"/ or Paraguay/ or Peru/ or Philippines/ or Romania/ or Russia/ or Rwanda/ or Saint Lucia/ or "Saint Vincent and the Grenadines"/ or "Independent State of Samoa"/ or "Sao Tome and Principe"/ or Senegal/ or Serbia/ or Sierra Leone/ or exp Melanesia/ or Sri Lanka/ or Somalia/ or South Sudan/ or Sudan/ or South Africa/ or Suriname/ or Swaziland/ or Syria/ or Tajikistan/ or Tanzania/ or Timor-Leste/ or Thailand/ or Togo/ or Tonga/ or Tunisia/ or Turkey/ or Turkmenistan/ or Uganda/ or Ukraine/ or Uzbekistan/ or Vanuatu/ or Venezuela/ or Vietnam/ or Yemen/ or Zambia/ or Zimbabwe/	1021632
25	(Afghanistan or Africa* or Albania* or Algeria* or American Samoa or Angola* or Argentin* or Armenia* or Azerbaijan* or Bangladesh* or Benin or Byelarus or Byelorussian or Belarus or Belorussian or Belorussia or Belize or Bhutan or Bolivia* or Bosnia* or Herzegovina or Hercegovina or Botswana or Brazil* or Brasil* or Bulgaria* or Burkina Faso or Burkina Fasso or Burundi or Urundi or Cambodia* or Khmer Republic or Kampuchea or Cameroon or Cameroons or Cameron or Camerons or Cape Verde or Cabo Verde or Chad or Colombia* or Comoros or Comoro Islands or Comores or Congo* or Costa Rica* or Cote d'Ivoire or Ivory Coast or Cuba* or Djibouti or Dominica or Dominican Republic or East Timor or East Timur or Timor Leste or Ecuador* or Egypt* or El Salvador* or Eritrea* or Equatorial Guinea* or Eswatini* or Ethiopia* or Fiji or Gabon or Gambia* or Gaza or Georgia Republic or Georgian Republic or Ghana* or Grenada or Guatemala* or Guinea* or Guyana* or Haiti* or Honduras or India* or Indonesia* or Iran* or Iraq* or Jamaica* or Jordan* or Kazakhstan* or Kenya* or Kiribati or Democratic People's Republic of Korea* or North Korea* or Kosovo or Kyrgyz Republic or Lao PDR or Laos or Lebanon or Lebanese or Lesotho or Liberia* or Libya* or Macedonia* or Madagascar or Malaysia* or Malaya* or Malay or Malawi or Mali or Maldives or Marshall Islands or Mauritania* or Mauritius or Mexic* or Mehico or Micronesia*	1731185

	or Middle East* or Moldova or Moldovia* or Moldovian* or Mongolia or Montenegro or Morocc* or Mozambique or Mocambique or Myanmar or Namibia* or Nauru or Nepal* or Nicaragua* or Niger or Nigeria* or Pakistan* or Palau or Palestin* or Paraguay or Peru* or Philippin* or Philipines or Phillipines or Phillippines or Romania* or Rumania* or Roumania* or Russia or Russian or Rwanda* or Ruanda* or Saint Lucia or St Lucia or Saint Vincent or St Vincent or Grenadines or Samoa or Samoan or Sao Tome or Senegal* or Serbia* or Sierra Leone or Spanish Guinea or Sri Lanka* or Ceylon or Solomon or Somalia* or Sudan* or Suriname or Surinam or Swaziland or Syria* or Tajikistan or Tadzhikistan or Tadjikistan or Tadzhik or Tanzania* or Thai* or Togo or Togolese or Tonga or Tunisia* or Turkey* or Turkiye* or Turkmenistan or Tuvalu or Uganda* or Ukrain* or Uzbekistan* or Uzbek or Vanuatu or Venezuela* or Vietnam* or Viet Nam or West Bank or Yemen* or Zambia* or Zimbabwe*).ti,ab,kf.	
26	(arab-countr* or arabic-countr* or middle-east* or sahara* or subsahara* or magreb* or maghrib* or west-indies* or caribbean* or central-america* or latin-america* or south-america* or central-asia* or north-asia* or northern-asia* or southeastern-asia* or south-eastern-asia* or southeast-asia* or south-east-asia* or west-asia* or western-asia* or east-europe* or eastern-europe*).ti,ab,kf.	195541
27	((developing or emerging or less* developed or under developed or underdeveloped or middle income or low* income or third-world or underserved or under served or deprived or poor*) adj3 (countr* or nation? or population? or world or economy or economies)).ti,ab,kf.	201703
28	((low* or middle*) adj5 (countr* or nation*)).ti,ab,kf.	78594
29	(low* adj2 (countr* or gdp or gnp or gross domestic or gross national)).ti,ab,kf.	19211
30	((rural or remote or nonmetropolitan or non-metropolitan or underserved or under served or deprived or shortage) adj (communit$ or count$ or area? or region? or province? or district?)).ti,ab,kf.	94167
31	(Global South or LIC or LMIC* or LMICs or MIC or South-South or rural health* or rural population*).ti,ab,kf.	95147
32	or/23-31	2288028
33	10 and 15 and 22 and 32	979

2. Embase

Interface: **embase.com** content coverage from 1947<br><br>Date of Search: 15 October 2024<br><br>Number of hits: 1,313<br><br>Comment: Emtree is the controlled vocabulary in Embase	Field labels<br><br>• /exp = exploded Emtree term<br>• /de = non exploded Emtree term<br>• ti,ab,kw = title, abstract and author keywords<br>• NEAR/x = within x words, regardless of order<br>• * = truncation of word for alternate endings<br>• $ = 0-1 letter/number<br>• ? = 1 letter/number

No.	Query	Results
#32	#30 NOT #31	1313
#31	#8 AND #13 AND #19 AND #29 AND [conference abstract]/lim	234
#30	#8 AND #13 AND #19 AND #29	1548
#29	#20 OR #21 OR #22 OR #23 OR #24 OR #25 OR #26 OR #27 OR #28	2931952
#28	'global south':ti,ab,kw OR lic:ti,ab,kw OR lmic*:ti,ab,kw OR lmics:ti,ab,kw OR mic:ti,ab,kw OR 'south south':ti,ab,kw OR 'rural health*':ti,ab,kw OR 'rural population*':ti,ab,kw	121667
#27	((rural OR remote OR nonmetropolitan OR 'non metropolitan' OR underserved OR 'under served' OR deprived OR shortage) NEXT/1 (communit? OR count? OR area$ OR region$ OR province$ OR district$)):ti,ab,kw	101527
#26	(low* NEAR/2 (countr* OR gdp OR gnp OR 'gross domestic' OR 'gross national')):ti,ab,kw	24085
#25	((low* OR middle*) NEAR/5 (countr* OR nation*)):ti,ab,kw	96261
#24	((developing OR emerging OR 'less* developed' OR 'under developed' OR underdeveloped OR 'middle income' OR 'low* income' OR 'third world' OR underserved OR 'under served' OR deprived OR poor*) NEAR/3 (countr* OR nation$ OR population$ OR world OR economy OR economies)):ti,ab,kw	220626

#23	'arab countr*':ti,ab,kw OR 'arabic countr*':ti,ab,kw OR 'middle east*':ti,ab,kw OR sahara*:ti,ab,kw OR subsahara*:ti,ab,kw OR magreb*:ti,ab,kw OR maghrib*:ti,ab,kw OR 'west indies*':ti,ab,kw OR caribbean*:ti,ab,kw OR 'central america*':ti,ab,kw OR 'latin america*':ti,ab,kw OR 'south america*':ti,ab,kw OR 'central asia*':ti,ab,kw OR 'north asia*':ti,ab,kw OR 'northern asia*':ti,ab,kw OR 'southeastern asia*':ti,ab,kw OR 'south eastern asia*':ti,ab,kw OR 'southeast asia*':ti,ab,kw OR 'south east asia*':ti,ab,kw OR 'west asia*':ti,ab,kw OR 'western asia*':ti,ab,kw OR 'east europe*':ti,ab,kw OR 'eastern europe*':ti,ab,kw	216261
#22	afghanistan:ti,ab,kw OR africa*:ti,ab,kw OR albania*:ti,ab,kw OR algeria*:ti,ab,kw OR 'american samoa':ti,ab,kw OR angola*:ti,ab,kw OR argentin*:ti,ab,kw OR armenia*:ti,ab,kw OR azerbaijan*:ti,ab,kw OR bangladesh*:ti,ab,kw OR benin:ti,ab,kw OR byelarus:ti,ab,kw OR byelorussian:ti,ab,kw OR belarus:ti,ab,kw OR belorussian:ti,ab,kw OR belorussia:ti,ab,kw OR belize:ti,ab,kw OR bhutan:ti,ab,kw OR bolivia*:ti,ab,kw OR bosnia*:ti,ab,kw OR herzegovina:ti,ab,kw OR hercegovina:ti,ab,kw OR botswana:ti,ab,kw OR brazil*:ti,ab,kw OR brasil*:ti,ab,kw OR bulgaria*:ti,ab,kw OR 'burkina faso':ti,ab,kw OR 'burkina fasso':ti,ab,kw OR burundi:ti,ab,kw OR urundi:ti,ab,kw OR cambodia*:ti,ab,kw OR 'khmer republic':ti,ab,kw OR kampuchea:ti,ab,kw OR cameroon:ti,ab,kw OR cameroons:ti,ab,kw OR cameron:ti,ab,kw OR camerons:ti,ab,kw OR 'cape verde':ti,ab,kw OR 'cabo verde':ti,ab,kw OR chad:ti,ab,kw OR colombia*:ti,ab,kw OR comoros:ti,ab,kw OR 'comoro islands':ti,ab,kw OR comores:ti,ab,kw OR congo*:ti,ab,kw OR 'costa rica*':ti,ab,kw OR 'cote d ivoire':ti,ab,kw OR 'ivory coast':ti,ab,kw OR cuba*:ti,ab,kw OR djibouti:ti,ab,kw OR dominica:ti,ab,kw OR 'dominican republic':ti,ab,kw OR 'east timor':ti,ab,kw OR 'east timur':ti,ab,kw OR 'timor leste':ti,ab,kw OR ecuador*:ti,ab,kw OR egypt*:ti,ab,kw OR 'el salvador*':ti,ab,kw OR eritrea*:ti,ab,kw OR 'equatorial guinea*':ti,ab,kw OR eswatini*:ti,ab,kw OR ethiopia*:ti,ab,kw OR fiji:ti,ab,kw OR gabon:ti,ab,kw OR gambia*:ti,ab,kw OR gaza:ti,ab,kw OR 'georgia republic':ti,ab,kw OR 'georgian republic':ti,ab,kw OR ghana*:ti,ab,kw OR grenada:ti,ab,kw OR guatemala*:ti,ab,kw OR guinea*:ti,ab,kw OR guyana*:ti,ab,kw OR haiti*:ti,ab,kw OR honduras:ti,ab,kw OR india*:ti,ab,kw OR indonesia*:ti,ab,kw OR iran*:ti,ab,kw OR iraq*:ti,ab,kw OR jamaica*:ti,ab,kw OR jordan*:ti,ab,kw OR kazakhstan*:ti,ab,kw OR kenya*:ti,ab,kw OR kiribati:ti,ab,kw OR 'democratic people* republic of korea*':ti,ab,kw OR 'north korea*':ti,ab,kw OR kosovo:ti,ab,kw OR 'kyrgyz republic':ti,ab,kw OR 'lao pdr':ti,ab,kw OR laos:ti,ab,kw OR lebanon:ti,ab,kw OR lebanese:ti,ab,kw OR lesotho:ti,ab,kw OR liberia*:ti,ab,kw OR libya*:ti,ab,kw OR macedonia*:ti,ab,kw OR madagascar:ti,ab,kw OR malaysia*:ti,ab,kw OR malaya*:ti,ab,kw OR malay:ti,ab,kw OR malawi:ti,ab,kw OR mali:ti,ab,kw OR maldives:ti,ab,kw OR 'marshall islands':ti,ab,kw OR mauritania*:ti,ab,kw OR mauritius:ti,ab,kw OR mexic*:ti,ab,kw OR mehico:ti,ab,kw OR micronesia*:ti,ab,kw OR 'middle east*':ti,ab,kw OR moldova:ti,ab,kw OR moldovia*:ti,ab,kw OR moldovian*:ti,ab,kw OR mongolia:ti,ab,kw OR montenegro:ti,ab,kw OR morocc*:ti,ab,kw OR mozambique:ti,ab,kw OR mocambique:ti,ab,kw OR myanmar:ti,ab,kw OR namibia*:ti,ab,kw OR nauru:ti,ab,kw OR nepal*:ti,ab,kw OR nicaragua*:ti,ab,kw OR niger:ti,ab,kw OR nigeria*:ti,ab,kw OR pakistan*:ti,ab,kw OR palau:ti,ab,kw OR palestin*:ti,ab,kw OR paraguay:ti,ab,kw OR peru*:ti,ab,kw OR philippin*:ti,ab,kw OR philipines:ti,ab,kw OR phillipines:ti,ab,kw OR phillippines:ti,ab,kw OR romania*:ti,ab,kw OR rumania*:ti,ab,kw OR roumania*:ti,ab,kw OR russia:ti,ab,kw OR russian:ti,ab,kw OR rwanda*:ti,ab,kw OR ruanda*:ti,ab,kw OR 'saint lucia':ti,ab,kw OR 'st lucia':ti,ab,kw OR 'saint vincent':ti,ab,kw OR 'st vincent':ti,ab,kw OR grenadines:ti,ab,kw OR samoa:ti,ab,kw OR samoan:ti,ab,kw OR 'sao tome':ti,ab,kw OR senegal*:ti,ab,kw OR serbia*:ti,ab,kw OR 'sierra leone':ti,ab,kw OR 'spanish guinea':ti,ab,kw OR 'sri lanka*':ti,ab,kw OR ceylon:ti,ab,kw OR solomon:ti,ab,kw OR somalia*:ti,ab,kw OR	2267942

	sudan*:ti,ab,kw OR suriname:ti,ab,kw OR surinam:ti,ab,kw OR swaziland:ti,ab,kw OR syria*:ti,ab,kw OR tajikistan:ti,ab,kw OR tadzhikistan:ti,ab,kw OR tadjikistan:ti,ab,kw OR tadzhik:ti,ab,kw OR tanzania*:ti,ab,kw OR thai*:ti,ab,kw OR togo:ti,ab,kw OR togolese:ti,ab,kw OR tonga:ti,ab,kw OR tunisia*:ti,ab,kw OR turkey*:ti,ab,kw OR turkiye*:ti,ab,kw OR turkmenistan:ti,ab,kw OR tuvalu:ti,ab,kw OR uganda*:ti,ab,kw OR ukrain*:ti,ab,kw OR uzbekistan*:ti,ab,kw OR uzbek:ti,ab,kw OR vanuatu:ti,ab,kw OR venezuela*:ti,ab,kw OR vietnam*:ti,ab,kw OR 'viet nam':ti,ab,kw OR 'west bank':ti,ab,kw OR yemen*:ti,ab,kw OR zambia*:ti,ab,kw OR zimbabwe*:ti,ab,kw	
#21	'afghanistan'/de OR 'albania'/de OR 'algeria'/de OR 'angola'/de OR 'argentina'/de OR 'armenia'/de OR 'azerbaijan'/de OR 'bangladesh'/de OR 'benin'/de OR 'belarus'/de OR 'belize'/de OR 'bhutan'/de OR 'bolivia'/de OR 'bosnia and herzegovina'/exp OR 'botswana'/de OR 'brazil'/exp OR 'bulgaria'/de OR 'burkina faso'/de OR 'burundi'/de OR 'cambodia'/de OR 'cameroon'/de OR 'cape verde'/de OR 'central african republic'/de OR 'chad'/de OR 'colombia'/de OR 'comoros'/de OR 'congo'/de OR 'costa rica'/de OR 'cote divoire' OR 'cuba'/de OR 'djibouti'/de OR 'dominican republic'/de OR 'ecuador'/de OR 'egypt'/de OR 'el salvador'/de OR 'eritrea'/de OR 'equatorial guinea'/de OR 'ethiopia'/de OR 'fiji'/de OR 'gabon'/de OR 'gambia'/de OR 'georgia (republic)'/exp OR 'ghana'/de OR 'grenada'/de OR 'guatemala'/de OR 'guinea'/de OR 'guinea bissau'/de OR 'guyana'/de OR 'haiti'/de OR 'honduras'/de OR 'india'/exp OR 'indonesia'/exp OR 'iran'/de OR 'iraq'/exp OR 'jamaica'/de OR 'jordan'/de OR 'kazakhstan'/de OR 'kenya'/de OR 'north korea'/de OR 'kosovo'/de OR 'kyrgyzstan'/de OR 'laos'/de OR 'lebanon'/de OR 'lesotho'/de OR 'liberia'/de OR 'libyan arab jamahiriya'/de OR 'republic of north macedonia'/de OR 'madagascar'/de OR 'malaysia'/exp OR 'malawi'/de OR 'mali'/de OR 'mauritania'/de OR 'federated states of micronesia'/de OR 'mauritius'/de OR 'mexico'/exp OR 'moldova'/de OR 'mongolia'/de OR 'montenegro (republic)'/de OR 'morocco'/de OR 'mozambique'/de OR 'myanmar'/de OR 'namibia'/de OR 'nepal'/de OR 'nicaragua'/de OR 'niger'/de OR 'nigeria'/de OR 'pakistan'/exp OR 'palau'/de OR 'papua new guinea'/de OR 'paraguay'/de OR 'peru'/de OR 'philippines'/de OR 'romania'/de OR 'russian federation'/exp OR 'rwanda'/de OR 'saint lucia'/de OR 'saint vincent and the grenadines'/de OR 'samoa'/de OR 'sao tome and principe'/de OR 'senegal'/de OR 'serbia'/exp OR 'sierra leone'/de OR 'melanesia'/de OR 'sri lanka'/de OR 'somalia'/exp OR 'south sudan'/de OR 'sudan'/de OR 'south africa'/de OR 'suriname'/de OR 'eswatini'/de OR 'syrian arab republic'/de OR 'tajikistan'/de OR 'tanzania'/de OR 'timor leste'/de OR 'thailand'/de OR 'togo'/de OR 'tonga'/de OR 'tunisia'/de OR 'turkey (republic)'/de OR 'turkmenistan'/de OR 'uganda'/de OR 'ukraine'/exp OR 'uzbekistan'/exp OR 'vanuatu'/de OR 'venezuela'/de OR 'viet nam'/de OR 'yemen'/de OR 'zambia'/de OR 'zimbabwe'/de	1415117
#20	'developing country'/exp OR 'developing country' OR 'low income country'/exp OR 'low income country' OR 'middle income country'/exp OR 'middle income country' OR 'rural health'/exp OR 'rural health' OR 'rural population'/exp OR 'rural population'	232724
#19	#14 OR #15 OR #16 OR #17 OR #18	3564568

#18	experience:ti,ab,kw OR experiences:ti,ab,kw OR perspective$:ti,ab,kw OR perception$:ti,ab,kw OR emotion$:ti,ab,kw OR feelings:ti,ab,kw OR attitude$:ti,ab,kw OR view$:ti,ab,kw	3169349
#17	'health behavior'/de OR 'attitude to health'/de OR 'health belief'/de	226798
#16	'emotion'/de	145261
#15	'attitude'/de OR 'health personnel attitude'/exp OR 'consumer attitude'/exp OR 'patient attitude'/de OR 'employee attitude'/de	375900
#14	'experience'/exp	43636
#13	#9 OR #10 OR #11 OR #12	2438943
#12	antibacterial:ti,ab,kw OR 'anti bacterial':ti,ab,kw OR antimicrobial:ti,ab,kw OR 'anti microbial':ti,ab,kw OR antibiotic*:ti,ab,kw OR 'anti biotic*':ti,ab,kw	923637
#11	'antibiotic resistance'/exp	240302
#10	'antimicrobial stewardship'/exp	12854
#9	'antibiotic agent'/exp	2044588
#8	#1 OR #2 OR #3 OR #4 OR #5 OR #6 OR #7	1017522
#7	multimethod*:ti,ab,kw OR 'multi method*':ti,ab,kw	6278
#6	((mixed OR multiple) NEAR/4 (design OR method* OR study)):ti,ab,kw	175561
#5	'focus group*':ti,ab,kw OR qualitative:ti,ab,kw OR ethnograph*:ti,ab,kw OR fieldwork:ti,ab,kw OR 'field work':ti,ab,kw OR 'key informant':ti,ab,kw	511989
#4	(('semi structured' OR semistructured OR unstructured OR informal OR 'in depth' OR indepth OR 'face to face' OR structured OR guide*) NEAR/3 (interview* OR discussion* OR questionnaire*)):ti,ab,kw	257473

#3	'ethnography'/exp	4244
#2	'qualitative research'/exp	135546
#1	'interview'/exp	404745

3. Cochrane Library

Interface: **Wiley** content coverage: - Cochrane Database of Systematic Reviews - April 1996<br>Central Trials - Current content July 1998<br><br>Date of Search: 15 October 2024<br><br>Number of hits: 254 (Reviews: 1, Trials: 253)	Field labels<br><br>• mh = exploded MeSH term<br>• mh ^= non exploded MeSH term<br>• ti,ab,kw = title, abstract and author keywords<br>• NEAR/x = within x words, regardless of order<br>• NEXT = used for truncated phrases<br>• NEXT/x = fixed word order<br>• * = truncation of word for alternate endings<br>• ? = 0-1 letter/number

ID Search Hits

#1 [mh "Interviews as topic"] 2556

#2 [mh "Focus groups"] 1085

#3 [mh Narration] 323

#4 [mh "Qualitative research"] 2408

#5 [mh "Personal narratives as topic"] 14

#6 ((semi-structured:ti,ab,kw OR semistructured:ti,ab,kw OR unstructured:ti,ab,kw OR informal:ti,ab,kw OR in-depth:ti,ab,kw OR indepth:ti,ab,kw OR face-to-face:ti,ab,kw OR structured:ti,ab,kw OR guide*:ti,ab,kw) NEAR/3 (interview*:ti,ab,kw OR discussion*:ti,ab,kw OR questionnaire*:ti,ab,kw)) 17363

#7 (("focus" NEXT group*):ti,ab,kw OR qualitative:ti,ab,kw OR ethnograph*:ti,ab,kw OR fieldwork:ti,ab,kw OR "field work":ti,ab,kw OR "key informant":ti,ab,kw) 28285

#8 ((mixed:ti,ab,kw OR multiple:ti,ab,kw) NEAR/4 (design:ti,ab,kw OR method*:ti,ab,kw OR study:ti,ab,kw)) 30403

#9 (multimethod*:ti,ab,kw OR multi-method*:ti,ab,kw) 798

#10 #1 OR #2 OR #3 OR #4 OR #5 OR #6 OR #7 OR #8 OR #9 67463

#11 [mh "Anti-Bacterial Agents"] 17114

#12 [mh ^"Antimicrobial Stewardship"] 123

#13 [mh "Drug resistance, microbial"] 3161

#14 (antibacterial:ti,ab,kw OR anti-bacterial:ti,ab,kw OR antimicrobial:ti,ab,kw OR anti-microbial:ti,ab,kw OR antibiotic*:ti,ab,kw OR anti-biotic*:ti,ab,kw) 52836

#15 #11 OR #12 OR #13 OR #14 55683

#16 [mh ^"Health Knowledge, Attitudes, Practice"] 8378

Karolinska Institutet

#17 [mh ^Attitude] 1616

#18 [mh ^"Attitude of health personnel"] 2811

#19 [mh ^"Attitude to health"] 3467

#20 [mh ^Emotions] 5360

#21 (experience:ti,ab,kw OR experiences:ti,ab,kw OR perspective?:ti,ab,kw OR perception?:ti,ab,kw OR emotion?:ti,ab,kw OR feelings:ti,ab,kw OR attitude?:ti,ab,kw OR view?:ti,ab,kw) 194276

#22 #16 OR #17 OR #18 OR #19 OR #20 OR #21 194276

#23 [mh ^"Medically Underserved Area"] OR [mh ^"Developing Countries"] OR [mh ^"Rural Health"] OR [mh ^"Rural Population"] 4840

#24 [mh ^Afghanistan] OR [mh ^Albania] OR [mh ^Algeria] OR [mh ^Angola] OR [mh ^Argentina] OR [mh ^Armenia] OR [mh ^Azerbaijan] OR [mh ^Bangladesh] OR [mh ^Benin] OR [mh ^"Republic of Belarus"] OR [mh ^Belize] OR [mh ^Bhutan] OR [mh ^Bolivia] OR [mh ^"Bosnia and Herzegovina"] OR [mh ^Botswana] OR [mh ^Brazil] OR [mh ^Bulgaria] OR [mh ^"Burkina Faso"] OR [mh ^Burundi] OR [mh ^Cambodia] OR [mh ^Cameroon] 5055

#25 [mh ^"Cabo Verde"] OR [mh ^"Central African Republic"] OR [mh ^Chad] OR [mh ^Colombia] OR [mh ^Comoros] OR [mh ^Congo] OR [mh ^"Costa Rica"] OR [mh ^"Cote d'Ivoire"] OR [mh ^Cuba] OR [mh ^Djibouti] OR [mh ^"Dominican Republic"] OR [mh ^Ecuador] OR [mh ^Egypt] OR [mh ^"El Salvador"] OR [mh ^Eritrea] OR [mh ^"Equatorial Guinea"] OR [mh ^Ethiopia] OR [mh ^Fiji] OR [mh ^Gabon] OR [mh ^Gambia] OR [mh ^Georgia] OR [mh ^Ghana] OR [mh ^Grenada] OR [mh ^Guatemala] 3449

#26 [mh ^Guinea] 21

#27 [mh ^Guinea-Bissau] 141

#28 [mh ^Guyana] OR [mh ^Haiti] OR [mh ^Honduras] OR [mh ^India] OR [mh ^Indonesia] OR [mh ^Iran] OR [mh ^Iraq] OR [mh ^Jamaica] OR [mh ^Jordan] 7331

#29 [mh ^Kazakhstan] OR [mh ^Kenya] OR [mh ^"Democratic People's Republic of Korea"] OR [mh ^Kosovo] OR [mh ^Kyrgyzstan] OR [mh ^Laos] OR [mh ^Lebanon] OR [mh ^Lesotho] OR [mh ^Liberia] OR [mh ^Libya] OR [mh ^Macedonia] OR [mh ^Madagascar] OR [mh ^Malaysia] OR [mh ^Malawi] OR [mh ^Mali] OR [mh ^Mauritania] OR [mh ^Micronesia] OR [mh ^Mauritius] OR [mh ^Mexico] OR [mh ^Moldova] OR [mh ^Mongolia] OR [mh ^Montenegro] OR [mh ^Morocco] OR [mh ^Mozambique] 4271

#30 [mh ^Myanmar] OR [mh ^Namibia] OR [mh ^Nepal] OR [mh ^Nicaragua] OR [mh ^Niger] OR [mh ^Nigeria] OR [mh ^Pakistan] OR [mh ^Palau] OR [mh ^"Papua New Guinea"] OR [mh ^Paraguay] OR [mh ^Peru] OR [mh ^Philippines] OR [mh ^Romania] OR [mh ^Russia] OR [mh ^Rwanda] OR [mh ^"Saint Lucia"] OR [mh ^"Saint Vincent and the Grenadines"] 3941

#31 [mh ^"Independent State of Samoa"] OR [mh ^"Sao Tome and Principe"] OR [mh ^Senegal] OR [mh ^Serbia] OR [mh ^"Sierra Leone"] OR [mh Melanesia] OR [mh ^"Sri Lanka"] OR [mh ^Somalia] OR [mh ^"South Sudan"] OR [mh ^Sudan] 795

#32 [mh ^"South Africa"] OR [mh ^Suriname] OR [mh ^Swaziland] 1984

#33 [mh ^Syria] OR [mh ^Tajikistan] OR [mh ^Tanzania] 1111

#34 [mh ^Timor-Leste] 9

#35 [mh ^Thailand] OR [mh ^Togo] OR [mh ^Tonga] OR [mh ^Tunisia] OR [mh ^Turkey] OR [mh ^Turkmenistan] OR [mh ^Uganda] OR [mh ^Ukraine] 4366

#36 [mh ^Uzbekistan] OR [mh ^Vanuatu] OR [mh ^Venezuela] OR [mh ^Vietnam] OR [mh ^Yemen] OR [mh ^Zambia] OR [mh ^Zimbabwe] 1468

#37 (Afghanistan:ti,ab,kw OR Africa*:ti,ab,kw OR Albania*:ti,ab,kw OR Algeria*:ti,ab,kw OR "American Samoa":ti,ab,kw OR Angola*:ti,ab,kw OR Argentin*:ti,ab,kw OR Armenia*:ti,ab,kw OR Azerbaijan*:ti,ab,kw OR Bangladesh*:ti,ab,kw OR Benin:ti,ab,kw OR Byelarus:ti,ab,kw OR Byelorussian:ti,ab,kw OR Belarus:ti,ab,kw OR Belorussian:ti,ab,kw OR Belorussia:ti,ab,kw OR Belize:ti,ab,kw OR Bhutan:ti,ab,kw OR Bolivia*:ti,ab,kw OR Bosnia*:ti,ab,kw OR Herzegovina:ti,ab,kw OR Hercegovina:ti,ab,kw OR Botswana:ti,ab,kw OR Brazil*:ti,ab,kw OR Brasil*:ti,ab,kw OR Bulgaria*:ti,ab,kw OR "Burkina Faso":ti,ab,kw OR "Burkina Fasso":ti,ab,kw OR Burundi:ti,ab,kw OR Urundi:ti,ab,kw OR Cambodia*:ti,ab,kw OR "Khmer Republic":ti,ab,kw OR Kampuchea:ti,ab,kw OR Cameroon:ti,ab,kw OR Cameroons:ti,ab,kw OR Cameron:ti,ab,kw OR Camerons:ti,ab,kw OR "Cape Verde":ti,ab,kw OR "Cabo Verde":ti,ab,kw OR Chad:ti,ab,kw OR Colombia*:ti,ab,kw OR Comoros:ti,ab,kw OR "Comoro Islands":ti,ab,kw OR Comores:ti,ab,kw OR Congo*:ti,ab,kw OR ("Costa" NEXT Rica*):ti,ab,kw OR "Cote d'Ivoire":ti,ab,kw OR "Ivory Coast":ti,ab,kw OR Cuba*:ti,ab,kw OR Djibouti:ti,ab,kw OR Dominica:ti,ab,kw OR "Dominican Republic":ti,ab,kw OR "East Timor":ti,ab,kw OR "East Timur":ti,ab,kw OR "Timor Leste":ti,ab,kw OR Ecuador*:ti,ab,kw OR Egypt*:ti,ab,kw OR ("El" NEXT Salvador*):ti,ab,kw OR Eritrea*:ti,ab,kw OR ("Equatorial" NEXT Guinea*):ti,ab,kw OR Eswatini*:ti,ab,kw OR Ethiopia*:ti,ab,kw OR Fiji:ti,ab,kw OR Gabon:ti,ab,kw OR Gambia*:ti,ab,kw OR Gaza:ti,ab,kw OR "Georgia Republic":ti,ab,kw OR "Georgian Republic":ti,ab,kw OR Ghana*:ti,ab,kw OR Grenada:ti,ab,kw OR Guatemala*:ti,ab,kw OR Guinea*:ti,ab,kw OR Guyana*:ti,ab,kw OR Haiti*:ti,ab,kw OR Honduras:ti,ab,kw OR India*:ti,ab,kw OR Indonesia*:ti,ab,kw OR Iran*:ti,ab,kw OR Iraq*:ti,ab,kw OR Jamaica*:ti,ab,kw OR Jordan*:ti,ab,kw OR Kazakhstan*:ti,ab,kw OR Kenya*:ti,ab,kw OR Kiribati:ti,ab,kw OR ("Democratic People's Republic of" NEXT Korea*):ti,ab,kw OR ("North" NEXT Korea*):ti,ab,kw OR Kosovo:ti,ab,kw OR "Kyrgyz Republic":ti,ab,kw OR "Lao PDR":ti,ab,kw OR Laos:ti,ab,kw OR Lebanon:ti,ab,kw OR Lebanese:ti,ab,kw OR Lesotho:ti,ab,kw OR Liberia*:ti,ab,kw OR Libya*:ti,ab,kw OR Macedonia*:ti,ab,kw OR Madagascar:ti,ab,kw OR Malaysia*:ti,ab,kw OR Malaya*:ti,ab,kw OR Malay:ti,ab,kw OR Malawi:ti,ab,kw OR Mali:ti,ab,kw OR Maldives:ti,ab,kw OR "Marshall Islands":ti,ab,kw OR Mauritania*:ti,ab,kw OR Mauritius:ti,ab,kw OR Mexic*:ti,ab,kw OR Mehico:ti,ab,kw OR Micronesia*:ti,ab,kw OR ("Middle" NEXT East*):ti,ab,kw OR Moldova:ti,ab,kw OR Moldovia*:ti,ab,kw OR Moldovian*:ti,ab,kw OR Mongolia:ti,ab,kw OR Montenegro:ti,ab,kw OR Morocc*:ti,ab,kw OR Mozambique:ti,ab,kw OR Mocambique:ti,ab,kw OR Myanmar:ti,ab,kw OR Namibia*:ti,ab,kw OR Nauru:ti,ab,kw OR Nepal*:ti,ab,kw OR Nicaragua*:ti,ab,kw OR Niger:ti,ab,kw OR Nigeria*:ti,ab,kw OR Pakistan*:ti,ab,kw OR Palau:ti,ab,kw OR Palestin*:ti,ab,kw OR Paraguay:ti,ab,kw OR Peru*:ti,ab,kw OR Philippin*:ti,ab,kw OR Philipines:ti,ab,kw OR Phillipines:ti,ab,kw OR Phillippines:ti,ab,kw OR Romania*:ti,ab,kw OR Rumania*:ti,ab,kw OR Roumania*:ti,ab,kw OR Russia:ti,ab,kw OR Russian:ti,ab,kw OR Rwanda*:ti,ab,kw OR Ruanda*:ti,ab,kw OR "Saint Lucia":ti,ab,kw OR "St Lucia":ti,ab,kw OR "Saint Vincent":ti,ab,kw OR "St Vincent":ti,ab,kw OR Grenadines:ti,ab,kw OR Samoa:ti,ab,kw OR Samoan:ti,ab,kw OR "Sao Tome":ti,ab,kw OR Senegal*:ti,ab,kw OR Serbia*:ti,ab,kw OR "Sierra Leone":ti,ab,kw OR "Spanish Guinea":ti,ab,kw OR ("Sri" NEXT Lanka*):ti,ab,kw OR Ceylon:ti,ab,kw OR Solomon:ti,ab,kw OR Somalia*:ti,ab,kw OR Sudan*:ti,ab,kw OR Suriname:ti,ab,kw OR Surinam:ti,ab,kw OR Swaziland:ti,ab,kw OR Syria*:ti,ab,kw OR Tajikistan:ti,ab,kw OR Tadzhikistan:ti,ab,kw OR Tadjikistan:ti,ab,kw OR Tadzhik:ti,ab,kw OR Tanzania*:ti,ab,kw OR Thai*:ti,ab,kw OR Togo:ti,ab,kw OR Togolese:ti,ab,kw OR Tonga:ti,ab,kw OR Tunisia*:ti,ab,kw OR Turkey*:ti,ab,kw OR Turkiye*:ti,ab,kw OR

Turkmenistan:ti,ab,kw OR Tuvalu:ti,ab,kw OR Uganda*:ti,ab,kw OR Ukrain*:ti,ab,kw OR Uzbekistan*:ti,ab,kw OR Uzbek:ti,ab,kw OR Vanuatu:ti,ab,kw OR Venezuela*:ti,ab,kw OR Vietnam*:ti,ab,kw OR "Viet Nam":ti,ab,kw OR "West Bank":ti,ab,kw OR Yemen*:ti,ab,kw OR Zambia*:ti,ab,kw OR Zimbabwe*:ti,ab,kw) 118353

#38 (arab-countr*:ti,ab,kw OR arabic-countr*:ti,ab,kw OR middle-east*:ti,ab,kw OR sahara*:ti,ab,kw OR subsahara*:ti,ab,kw OR magreb*:ti,ab,kw OR maghrib*:ti,ab,kw OR west-indies*:ti,ab,kw OR caribbean*:ti,ab,kw OR central-america*:ti,ab,kw OR latin-america*:ti,ab,kw OR south-america*:ti,ab,kw OR central-asia*:ti,ab,kw OR north-asia*:ti,ab,kw OR northern-asia*:ti,ab,kw OR southeastern-asia*:ti,ab,kw OR south-eastern-asia*:ti,ab,kw OR southeast-asia*:ti,ab,kw OR south-east-asia*:ti,ab,kw OR west-asia*:ti,ab,kw OR western-asia*:ti,ab,kw OR east-europe*:ti,ab,kw OR eastern-europe*:ti,ab,kw) 7305

#39 ((developing:ti,ab,kw OR emerging:ti,ab,kw OR (less* NEXT "developed"):ti,ab,kw OR "under developed":ti,ab,kw OR underdeveloped:ti,ab,kw OR "middle income":ti,ab,kw OR (low* NEXT "income"):ti,ab,kw OR third-world:ti,ab,kw OR underserved:ti,ab,kw OR "under served":ti,ab,kw OR deprived:ti,ab,kw OR poor*:ti,ab,kw) NEAR/3 (countr*:ti,ab,kw OR nation?:ti,ab,kw OR population?:ti,ab,kw OR world:ti,ab,kw OR economy:ti,ab,kw OR economies:ti,ab,kw)) 11291

#40 ((low*:ti,ab,kw OR middle*:ti,ab,kw) NEAR/5 (countr*:ti,ab,kw OR nation*:ti,ab,kw)) 5973

#41 (low*:ti,ab,kw NEAR/2 (countr*:ti,ab,kw OR gdp:ti,ab,kw OR gnp:ti,ab,kw OR "gross domestic":ti,ab,kw OR "gross national":ti,ab,kw)) 1730

#42 ((rural:ti,ab,kw OR remote:ti,ab,kw OR nonmetropolitan:ti,ab,kw OR non-metropolitan:ti,ab,kw OR underserved:ti,ab,kw OR "under served":ti,ab,kw OR deprived:ti,ab,kw OR shortage:ti,ab,kw) NEXT (communit?:ti,ab,kw OR count?:ti,ab,kw OR area?:ti,ab,kw OR region?:ti,ab,kw OR province?:ti,ab,kw OR district?:ti,ab,kw)) 4844

#43 ("Global South":ti,ab,kw OR LIC:ti,ab,kw OR LMIC*:ti,ab,kw OR LMICs:ti,ab,kw OR MIC:ti,ab,kw OR South-South:ti,ab,kw OR ("rural" NEXT health*):ti,ab,kw OR ("rural" NEXT population*):ti,ab,kw) 7739

#44 #23 OR #24 OR #25 OR #26 OR #27 OR #28 OR #29 OR #30 OR #31 OR #32 OR #33 OR #34 OR #35 OR #36 OR #37 OR #38 OR #39 OR #40 OR #41 OR #42 OR #43 131930

#45 #10 AND #15 AND #44 254

4. Web of Science Core Collection

Interface: **Clarivate Analytics**

Editions and content coverage years= A&HCI - 1975 , ESCI -2019 , SCI-EXPANDED - 1945 , SSCI - 1945

Date of Search: 15 October 2024

Number of hits: 662

Field labels

- TS/Topic = title, abstract, author keywords and Keywords Plus
- TI= title
- AB = abstract
- AK = author keywords
- NEAR/x = within x words, regardless of order
- * = truncation of word for alternate endings
- # = 0-1 letter/number
- ? = 1 letter/number

Note: the *Exact search*-function was used for all the searches

#	Search Query	Results
1	TS=((semi-structured OR semistructured OR unstructured OR informal OR in-depth OR indepth OR face-to-face OR structured OR guide*) NEAR/3 (interview* OR discussion* OR questionnaire*))	299653
2	TS=("focus group*" OR qualitative OR ethnograph* OR fieldwork OR "field work" OR "key informant")	838674
3	TS=((mixed OR multiple) NEAR/4 (design OR method* OR study))	296754
4	TS=(multimethod* OR multi-method*)	13012
5	#4 OR #3 OR #2 OR #1	1250291
6	TS=(antibacterial OR anti-bacterial OR antimicrobial OR anti-microbial OR antibiotic* OR anti-biotic*)	896022
7	TS=(experience OR experiences OR perspective$ OR perception$ OR emotion$ OR feelings OR attitude$ OR view$)	4809681
8	TS=(Afghanistan OR Africa* OR Albania* OR Algeria* OR "American Samoa" OR Angola* OR Argentin* OR Armenia* OR Azerbaijan* OR Bangladesh* OR Benin OR Byelarus OR Byelorussian OR Belarus OR Belorussian OR Belorussia OR Belize OR Bhutan OR Bolivia* OR Bosnia* OR Herzegovina OR Hercegovina OR Botswana OR Brazil* OR Brasil* OR Bulgaria* OR "Burkina Faso" OR "Burkina Fasso" OR Burundi OR Urundi OR Cambodia* OR "Khmer Republic" OR Kampuchea OR Cameroon OR Cameroons OR Cameron OR Camerons OR "Cape Verde" OR "Cabo Verde" OR Chad OR Colombia* OR Comoros OR "Comoro Islands" OR Comores OR Congo* OR "Costa Rica*" OR "Cote d'Ivoire" OR "Ivory Coast" OR Cuba* OR Djibouti OR Dominica OR "Dominican Republic" OR "East Timor" OR "East Timur" OR "Timor Leste" OR Ecuador* OR Egypt* OR "El Salvador*" OR Eritrea* OR "Equatorial Guinea*" OR Eswatini* OR Ethiopia* OR Fiji OR Gabon OR Gambia* OR Gaza OR "Georgia Republic" OR "Georgian Republic" OR Ghana* OR Grenada OR Guatemala* OR	4248781

	Guinea* OR Guyana* OR Haiti* OR Honduras OR India* OR Indonesia* OR Iran* OR Iraq* OR Jamaica* OR Jordan* OR Kazakhstan* OR Kenya* OR Kiribati OR "Democratic People's Republic of Korea*" OR "North Korea*" OR Kosovo OR "Kyrgyz Republic" OR "Lao PDR" OR Laos OR Lebanon OR Lebanese OR Lesotho OR Liberia* OR Libya* OR Macedonia* OR Madagascar OR Malaysia* OR Malaya* OR Malay OR Malawi OR Mali OR Maldives OR "Marshall Islands" OR Mauritania* OR Mauritius OR Mexic* OR Mehico OR Micronesia* OR "Middle East*" OR Moldova OR Moldovia* OR Moldovian* OR Mongolia OR Montenegro OR Morocc* OR Mozambique OR Mocambique OR Myanmar OR Namibia* OR Nauru OR Nepal* OR Nicaragua* OR Niger OR Nigeria* OR Pakistan* OR Palau OR Palestin* OR Paraguay OR Peru* OR Philippin* OR Philipines OR Phillipines OR Phillippines OR Romania* OR Rumania* OR Roumania* OR Russia OR Russian OR Rwanda* OR Ruanda* OR "Saint Lucia" OR "St Lucia" OR "Saint Vincent" OR "St Vincent" OR Grenadines OR Samoa OR Samoan OR "Sao Tome" OR Senegal* OR Serbia* OR "Sierra Leone" OR "Spanish Guinea" OR "Sri Lanka*" OR Ceylon OR Solomon OR Somalia* OR Sudan* OR Suriname OR Surinam OR Swaziland OR Syria* OR Tajikistan OR Tadzhikistan OR Tadjikistan OR Tadzhik OR Tanzania* OR Thai* OR Togo OR Togolese OR Tonga OR Tunisia* OR Turkey* OR Turkiye* OR Turkmenistan OR Tuvalu OR Uganda* OR Ukrain* OR Uzbekistan* OR Uzbek OR Vanuatu OR Venezuela* OR Vietnam* OR "Viet Nam" OR "West Bank" OR Yemen* OR Zambia* OR Zimbabwe*)	
9	TS=(arab-countr* OR arabic-countr* OR middle-east* OR sahara* OR subsahara* OR magreb* OR maghrib* OR west-indies* OR caribbean* OR central-america* OR latin-america* OR south-america* OR central-asia* OR north-asia* OR northern-asia* OR southeastern-asia* OR south-eastern-asia* OR southeast-asia* OR south-east-asia* OR west-asia* OR western-asia* OR east-europe* OR eastern-europe*)	474484
10	TS=((developing OR emerging OR "less* developed" OR "under developed" OR underdeveloped OR "middle income" OR "low* income" OR third-world OR underserved OR "under served" OR deprived OR poor*) NEAR/3 (countr* OR nation$ OR population$ OR world OR economy OR economies))	319878
11	TS=((low* OR middle*) NEAR/5 (countr* OR nation*))	111329
12	TS=(low* NEAR/2 (countr* OR gdp OR gnp OR "gross domestic" OR "gross national"))	39509
13	TS=((rural OR remote OR nonmetropolitan OR non-metropolitan OR underserved OR "under served" OR deprived OR shortage) NEAR/0 (communit? OR count? OR area$ OR region$ OR province$ OR district$))	117754
14	TS=("Global South" OR LIC OR LMIC* OR LMICs OR MIC OR South-South OR "rural health*" OR "rural population*")	120872
15	#8 OR #9 OR #10 OR #11 OR #12 OR #13 OR #14	477685<br>0

16	#15 AND #7 AND #6 AND #5	662

Karolinska Institutet

5. Google Scholar

Interface: Publish or perish<br><br>Date of Search: 15 October 2024<br><br>Number of hits: 200	| = OR
antibacterial|anti-bacterial|antimicrobial|anti-microbial|antibiotic|anti-biotic LMIC|LMICs|”developing country”|”low income”|”middle income” experience|perspective|perception|attitude|view	
